# Tuning riboflavin derivatives for photodynamic inactivation of pathogens

**DOI:** 10.1038/s41598-022-10394-7

**Published:** 2022-04-21

**Authors:** Leander B. Crocker, Ju Hyun Lee, Suraj Mital, Gabrielle C. Mills, Sina Schack, Andrea Bistrović-Popov, Christoph O. Franck, Ioanna Mela, Clemens F. Kaminski, Graham Christie, Ljiljana Fruk

**Affiliations:** Department of Chemical Engineering and Biotechnology, University of Cambridge, Philippa Fawcett Drive, Cambridge, CB3 0AS UK

**Keywords:** Chemical synthesis, Photochemistry, Bacterial infection, Viral infection

## Abstract

The development of effective pathogen reduction strategies is required due to the rise in antibiotic-resistant bacteria and zoonotic viral pandemics. Photodynamic inactivation (PDI) of bacteria and viruses is a potent reduction strategy that bypasses typical resistance mechanisms. Naturally occurring riboflavin has been widely used in PDI applications due to efficient light-induced reactive oxygen species (ROS) release. By rational design of its core structure to alter (photo)physical properties, we obtained derivatives capable of outperforming riboflavin’s visible light-induced PDI against *E. coli* and a SARS-CoV-2 surrogate, revealing functional group dependency for each pathogen. Bacterial PDI was influenced mainly by guanidino substitution, whereas viral PDI increased through bromination of the flavin. These observations were related to enhanced uptake and ROS-specific nucleic acid cleavage mechanisms. Trends in the derivatives’ toxicity towards human fibroblast cells were also investigated to assess viable therapeutic derivatives and help guide further design of PDI agents to combat pathogenic organisms.

## Introduction

Riboflavin and its derivatives, flavin mononucleotide (FMN) and flavin adenine dinucleotide (FAD) are organic cofactors found within enzymes involved in numerous biochemical pathways^1,2^. Due to their rich redox chemistry and ability to mediate a wide range of both oxidative and reductive organic transformations, flavin-containing enzymes are prominently used as biocatalysts^3,4^. Flavins are also involved in the regulation of photochemical pathways due to strong blue light absorption, which results in the generation of highly oxidising excited states that can elicit biological signalling events or responses^5–8^. This photoexcitation process has been exploited in a number of photocatalytic applications, whereby modification of the flavin chromophore can enable the formation of high potential oxidative and reductive intermediates to afford useful synthetic methodology^9–11^.

Another application in which photoexcitation is utilised to generate reactive intermediates is within photodynamic inactivation (PDI) of pathogens. In this technique, a photosensitiser (PS) such as riboflavin, is employed to generate a burst of reactive oxygen species (ROS) in the vicinity of a particular pathogen in order to cause irreversible damage that leads to inactivation^12,13^. Due to the rapid onset and non-specificity of PS-induced ROS release, conventional mechanisms of pathogen resistance through specific efflux pumps or detoxification pathways can be avoided^14^. Therefore, PDI of pathogens is a versatile and effective strategy that has the potential to help combat increasing antibiotic-resistant bacteria strains^14,15^, and ongoing zoonotic viral pandemics^16^.

**Figure 1:**
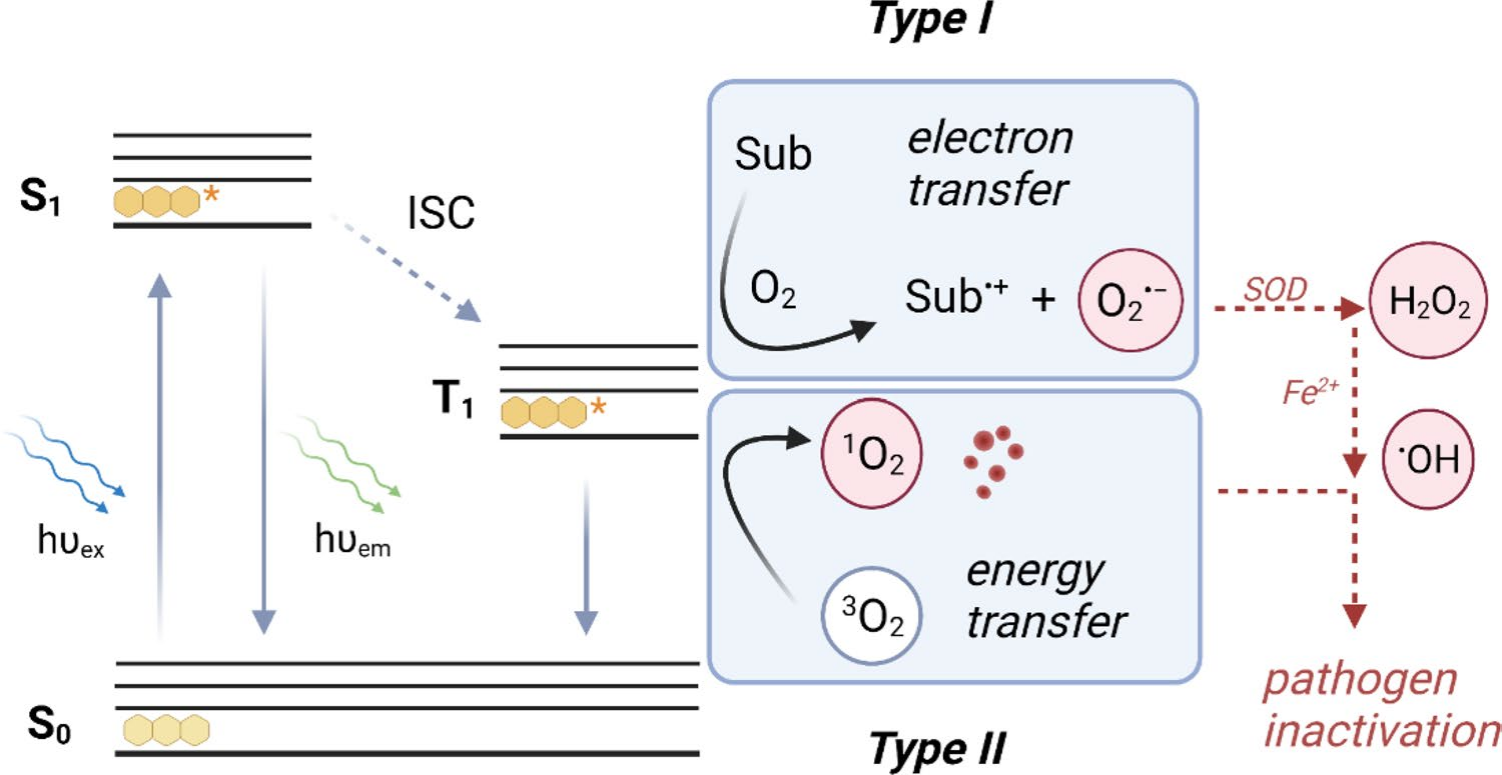
Mechanism of photosensitised reactive oxygen species (ROS) formation that enables inactivation of pathogens. ISC = intersystem crossing, Sub = substrate, SOD = superoxide dismutase.

Mechanistically, PDI of a pathogen is initiated by excitation of a PS with a suitable wavelength of light (hν_ex_) which generates an excited singlet state (S_1_). This excited state can either fluoresce (hν_em_) back to its ground state (S_0_) or undergo intersystem crossing (ISC) to an excited triplet state (T_1_), typically characterised by a longer lifetime (Fig. 1). This excited triplet state PS can interact with substrate such as an amino acid residue or nucleobase to form an oxidised substrate and reduced PS that subsequently reduces oxygen to superoxide (O_2_^•-^) (Type I reaction, Fig. 1)^17^. Superoxide is produced under oxidative stress naturally and is an important factor of cell signalling pathways. Both prokaryotic and eukaryotic cells contain superoxide dismutase (SOD) enzymes in order to mitigate superoxide’s cytotoxic effects^17^. Other types of ROS can be formed in Type I reactions such as H_2_O_2_ and hydroxyl radicals (^•^OH), however catalase and glutathione can neutralise these species respectively^17^.

Type II reactions lead to the formation of singlet oxygen (^1^O_2_), a highly cytotoxic ROS that non-selectively oxidises biomolecules such as nucleic acids, proteins and lipids in order to inactivate the pathogen^17^. Singlet oxygen formation occurs through the interaction of the excited triplet state PS with ground state triplet oxygen (^3^O_2_, Fig. 1). The efficiency of the light absorption that leads to this event can be quantified through the measurement of the singlet oxygen quantum yield (Ф_Δ_). Effective defence mechanisms against singlet oxygen have not evolved within non-photosynthetic microorganisms, hence it is considered key to PDI of bacterial and viral pathogens^12,13,18^.

Due to its excellent photosensitising properties, abundance and lack of toxicity, riboflavin has several applications within blood product sterilisation in combination with ultraviolet (UV) light for effective PDI of pathogens prior to transfusion^19,20^. More recently, it was shown to inactivate coronaviruses such as MERS and SARS-CoV-2^21–23^. Similarly, it has been reported that riboflavin and FMN can inactivate bacteria^24^, cancer cells^25^ and parasites^26^ using either UV or blue (440–460 nm) light. Despite such promising studies, the design of flavin derivatives to understand structure–activity relationships towards PDI applications has not been explored, unlike prominent photosensitisers such as porphyrins^27^. Previously, it was shown that functionalisation of flavins with amino groups, which introduce positive charge, can enhance PDI against both Gram-negative and Gram-positive bacteria as well as endospores^28,29^. In general, cationic groups are known to improve the efficacy of PDI by coordination of the molecule to negatively charged phospholipid membranes. However, charge is not the only factor that contributes to the efficiency of PDI. For example, lipophilic flavin derivatives bearing multiple acetyl ester moieties have been shown to improve the efficacy against the *Leishmania major* parasite over analogous cationic equivalents, highlighting the importance of the cell membrane permeability^26^.

**Figure 2:**
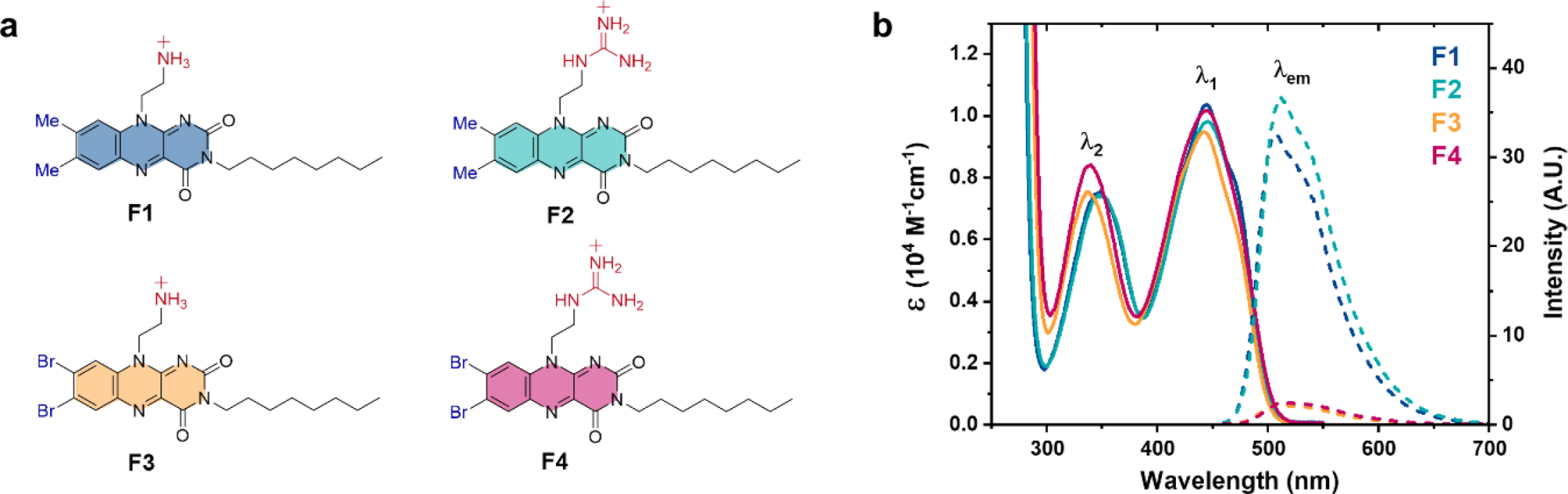
The structures and photophysical spectra of **F1-4**. (**a**) Structures of flavins **F1-4** (counteranions = TFA^-^), (**b**) UV–Vis absorption (bold) and emission (dashed) spectra of **F1-4** in DMSO.

By considering the mechanism of PDI, as well as the biochemical structure of pathogens, we report the rational design of flavin derivatives (**F1-4**, Fig. 2a) for pathogen inactivation. The prepared flavins demonstrate high efficacy and rapid inactivation of two model pathogens, a Gram-negative bacterium, *E. coli* BL21(DE3), and, murine hepatitis virus A59 strain (MHV-A59)^30^ often used as a surrogate for study of other coronaviruses such as SARS-Cov-2 despite the differences in transmission path, under white light irradiation (400–700 nm). In addition, the toxicity of the derivatives towards human fibroblast cells (WI-38) is explored, revealing that clear structure–activity relationships can be observed relating to the functionality of derivatives.

## Results

### Synthesis and characterisation of flavins

Flavins **F1-4** (**2A**) were prepared according to Schemes S1 and S2 (see ESI). Inspired by the classical amphiflavins developed by Trissel, Schmidt and Hemmerich^31–33^, we chose to include an alkyl chain (C_8_) within the structure to improve phospholipid membrane incorporation. Additionally, the flavin chromophore itself was substituted with bromo groups in the case of **F3-4** in order to harness the heavy-atom effect that increases the rate of ISC from singlet to triplet excited states, thereby potentially enhancing photosensitised generation of singlet oxygen^34^. Heavy atom substitution has been shown to improve both the rate of ISC and the singlet oxygen quantum yield (Ф_Δ_) in flavin derivatives used for synthetic photooxidation reactions^35^. Although the heavy-atom effect has been used to boost photodynamic efficacy for other PS dyes in PDI applications, it has not yet been explored for flavin derivatives ^36^.

To afford both methylated (**F1-2**) and brominated derivatives (**F3-4**), a Boc-protected ethylene amino component was first installed to the methylated or brominated arene core prior to cyclisation of the isoalloxazine ring system. Following N^3^-alkylation with an octyl chain on the isoalloxazine, N-Boc protecting groups were removed to yield the amino-functionalised **F1** or **F3** that were subsequently converted to guanidino moieties to achieve **F2** or **F4** respectively (Fig. 2a). Guanidino groups were chosen as they are known to increase membrane coordination and penetration through strong guanidinium-phosphate H-bonding and we hypothesised would enhance PDI efficacy.

The UV–Vis absorption spectra of the flavins in DMSO revealed very similar absorption properties at the λ_1_ (S_0_ → S_1_) band but a blue shift of 10–13 nm for the higher energy λ_2_ (S_0_ → S_2_) bands of the brominated compounds (**F3** and **F4**) presumably due to the electron withdrawing effect of Br atoms (Fig. 2b, Table 1, Table S1). In terms of emission properties, the heavy-atom effect of bromination can be clearly observed with **F3** and **F4** exhibiting severely reduced emission intensity (Ф_F_ < 2% in DMSO, Table 1) when compared to the methylated **F1** and **F2** (Ф_F_ = 13% and 14% respectively in DMSO, Table 1). This effect can also be observed when comparing the efficiency of ^1^O_2_ production upon excitation, where brominated **F3** and **F4** demonstrate up to a 30% increase in activity over the methylated **F1** and **F2** in MeCN (Table 1), indicating their potential to be potent photodynamic agents. Despite this, **F1** and **F2** are still efficient ^1^O_2_ photosensitisers comparable with riboflavin (Ф_Δ_ = 0.54 ± 0.07). However, a smaller Ф_Δ_ value for guanidinylated **F4** (62%) is observed compared to aminated **F3** (85%) which could be explained by fast reverse ISC and/or solvent-dependent aggregation.

**Table 1:** Photophysical properties of flavins **F1-4**. λ_n_ = S_0_ → S_n_ absorption band, λ_em_ = emission wavelength, φ_F_ = fluorescence quantum yield, φ_Δ_ = singlet oxygen quantum yield.

Flavin	λ_1_ (nm)^a^	λ_2_ (nm)^a^	λ_em_ (nm)^a^	φ_F_^b^	φ_Δ_^c^
F1	444	349	507	0.129 ± 0.012	0.49 ± 0.12
F2	446	350	512	0.141 ± 0.010	0.55 ± 0.10
F3	443	336	508	0.009 ± 0.013	0.85 ± 0.09
F4	444	340	513	0.011 ± 0.010	0.62 ± 0.10

^a^measured in DMSO.

^b^calculated using riboflavin as the reference (ϕ_F_ = 0.226 ± 0.001 in DMSO).

^c^calculated using Ru(bpy)_3_^2+^ as the reference (ϕ_Δ_ = 0.57 ± 0.06 in MeCN).

After successful synthesis and initial characterisation, we monitored the photostability of the compounds in PBS (1x, pH 7.4) which was used for PDI assays as distilled or ultrapure water destabilises bacterial cells and coronaviruses through osmotic pressures^37,38^, thereby augmenting inactivation results. Flavins **F1-4** and riboflavin (100 µM in PBS) were therefore irradiated with a 6200 K white LED light source (18 W, 400–700 nm, see ESI Figure S1 for emission spectrum) at an illuminance of 1 × 10^5^ lx (35 mW/cm^2^ irradiance) to resemble typical daylight ^39^, and the changes in their UV–Vis absorption were monitored over time (see ESI, Figure S4). For riboflavin, rapid photodecomposition (80%) was observed over 30 min irradiation which is known to be due to intramolecular dealkylation of the ribityl chain (Figure S4a,b). This yields lumichrome as the major degradation product which can only act as a PS under UV-irradiation^40^. Amino-containing **F1** and **F3** exhibited around 40% and 60% degradation respectively after the same irradiation time (Figure S4a,c,e), whereas **F2** and **F4** degraded by approximately 10% and 30% respectively (Figure S4a,d,f.). These findings corroborate previous work showing photodegradation of amino-containing flavins in the presence of phosphate ions, which resulted in diminished bacterial PDI efficiency^41^. However, it appears that guanidino substitution in our case improves the photostability. Interestingly, bromo-substituted flavins show higher rates of photodegradation, which could be explained by their higher Ф_Δ_ values resulting in greater ^1^O_2_-induced degradation.

### Photodynamic inactivation of *E. coli*

**Figure 3:**
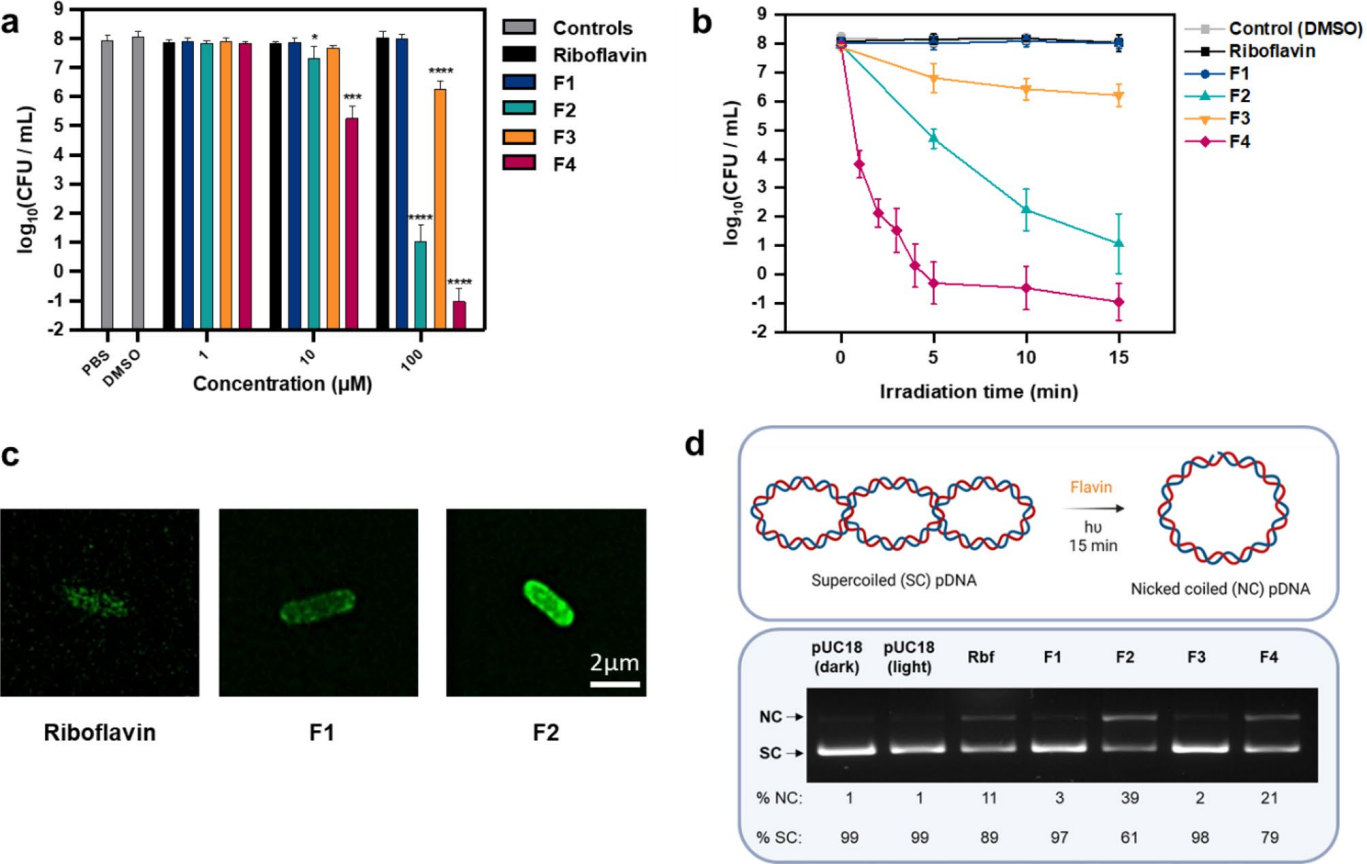
Photodynamic inactivation of *E. coli*. (**a**) Photodynamic inactivation of *E. coli* BL21(DE3) incubated with various concentrations of flavins irradiated with white LED light (1 × 10^5^ lx, 35 mW/cm^2^) for 15 min in PBS containing 0.5% DMSO. Data are expressed as the mean ± SD of three biological replicates. Significance levels are defined compared to PBS control as the following: * for p ≤ 0.05, ** for p ≤ 0.01, *** for p < 0.001, and **** for p < 0.0001 (**b**) Time course of *E. coli* inactivation incubated with 100 µM of flavin irradiated with white LED light (1 × 10^5^ lx, 35 mW/cm^2^) for 15 min in PBS containing 0.5% DMSO. Data are expressed as the mean ± RE of three biological replicates. (**c**) SIM images depicting the localisation of flavins in *E. coli* after incubation with 100 µM flavin. (**d**) Agarose gel electrophoresis of pDNA (pUC18) following incubation with 10 µM flavin irradiated with white LED light (1 × 10^5^ lx, 35 mW/cm^2^) for 15 min in PBS containing 0.1% DMSO. Rbf = Riboflavin. Uncropped gels can be found in ESI (Figure S9).

Having observed clear trends in the photophysical properties of methylated and brominated derivatives, we were interested to see how this would affect the PDI efficacy of **F1-4** against pathogens. First, we investigated the inactivation of the Gram-negative bacterium, *E. coli* BL21(DE3)*.* The cell envelope of Gram-negative bacteria presents a formidable barrier to antimicrobial compounds that consequently inhibits PDI efficacy compared to the analogous structure in Gram-positive bacteria^42^. Following an initial 20 min incubation of the flavin compounds with *E. coli* in PBS at various concentrations in the dark, the mixture was irradiated (1 × 10^5^ lx) and the number of surviving colony forming units (CFUs) were determined. After 15 min (31.5 J/cm^2^ light dose) of irradiation, no inactivation was observed at 1 µM for either riboflavin or **F1-4**. At 10 µM approximately 1 log_10_ reduction of *E. coli* CFUs was observed for **F2** and a 2.8 log_10_ reduction in the presence of **F4** (Fig. 3a, Table S2). At higher concentrations, this effect was greatly enhanced with both **F2** and **F4** exhibiting > 6.0 log_10_ reduction of CFU/mL at 100 µM which is considered a highest level of decontamination according to EMA guidance^43^.

Interestingly, riboflavin and **F1** demonstrated no activity at 100 µM, whereas **F3** exhibited a 1.8 log_10_ reduction in bacterial load. To ensure effective PDI was occurring, the flavin compounds were incubated in the dark under the same experimental irradiation conditions (100 µM, 15 min) to reveal no bacterial toxicity (see ESI, Figure S5). The rate of *E. coli* inactivation over time was then monitored at a 100 µM flavin concentration revealing an extremely rapid reduction of CFUs in the presence of **F4**, facilitating > 6.0 log_10_ reduction after just 5 min of irradiation (10.5 J/cm^2^ light dose). Similarly, **F2** shows effective bactericidal activity with a 3.4 log_10_ reduction (> 99.9%) after 5 min irradiation. A closer investigation into the speed of **F4**’s activity at 100 µM showed that after only 1 min of irradiation (2.10 J/cm^2^ light dose) a 4.1 log_10_ reduction (> 99.99%) of bacteria was achieved (Fig. 3b).

These data clearly show that the introduction of a guanidino moiety, as in the case of **F2** and **F4**, greatly increases the flavin’s PDI efficacy against the bacterium when compared to amino-containing **F1** and **F3**. This may be attributed to improved photostability in PBS, as well as the guanidino group facilitating better coordination to the cytoplasmic phospholipid membrane resulting in improved permeability and lipid peroxidation under irradiation^44^. In addition, the replacement of methyl substituents with bromines improves efficacy when the same cationic group is compared. This can be explained by more efficient generation of singlet oxygen, as predicted by their Ф_Δ_ values shown in Table 1. As a result, greater lipid and biomolecule oxidation can be achieved to inactivate the pathogen. It should also be noted that the inclusion of bromine atoms increases the lipophilicity of the molecule which can further improve cell membrane permeability and incorporation. However, despite having the highest predicted Ф_Δ_ value (85%), **F3** did not outperform **F2** (Ф_Δ_ = 55%) which demonstrates the overarching impact of guanidino substitution.

To gain initial mechanistic insight, we investigated the cellular localisation of fluorescent riboflavin, **F1** and **F2** by structured illumination microscopy (SIM) (Fig. 3c, see ESI Figures S6,, S7, S8). After incubation with the flavin compounds (100 µM), large field of view images revealed marked differences in the number and degree of fluorescent bacterial populations (see ESI Figures S6, S7, S8). Although the exact quantification of flavin uptake was not possible due to uncharacterised optical properties in a complex biological environment, the qualitative comparison of **F2** and **F1** (which have similar Ф_F_ in DMSO), revealed a higher number of fluorescent bacteria with bright fluorescent intensity after incubation with **F2**, especially within membranes (Fig. 3c, Figure S6 and S7). In addition, two subpopulations of bacteria can be observed, one in which intense flavin fluorescence is seen in the cell envelope and in the cytosolic space, and one in which fluorescence is seen only in the cell envelope and not in the cytosolic space. We believe that the existence of these two subpopulations indicate a level of cytosolic uptake in some cells. Although bacterial membranes serve as an excellent barrier for uptake of extracellular compounds, it is plausible that produced singlet oxygen might damage the integrity of bacterial envelope easing the penetration of flavin compounds. It can also be observed that guanidino substitution seems to lead to an enhanced uptake in comparison with other compounds. However, the distribution of fluorescent intensity was not homogeneous across different bacterial cells which could be related to the amphiphilicity of **F2**, resulting in variable uptake due to aggregation (Figure S6). For hydrophilic riboflavin, very weak fluorescent populations were observed, most likely explained by the controlled transport of the compound through outer membrane porins of the bacterium and therefore unable to bind effectively to the outer or cytoplasmic membranes (Fig. 3c, Figure S8). This therefore helps to explain the lack of PDI efficacy observed when using riboflavin.

Enhanced PDI efficacy of flavins that seem to be taken up into the cytosol can be explained by their ability to interact with biomolecules within the cell, such as nucleic acid. To test this hypothesis, we monitored the pDNA (pUC18) cleavage in the presence of the flavins (10 μM) under irradiation, which indicated guanidino derivatives possess superior photocleavage ability (Fig. 3d). The photocleavage of supercoiled (SC) to nicked coiled (NC) pDNA structures was significantly enhanced for **F2** and **F4** (39% and 21% respectively) when compared to **F1** and **F3** (≤ 3%) after irradiation for 15 min (Fig. 3d). This difference in activity could be explained by favourable guanidinium-phosphate interactions that increase the likelihood of electron transfer events between flavin and guanosine, which are known to primarily contribute to DNA cleavage alongside ^1^O_2_-mediated oxidation^45,46^. As a consequence, even if amino-flavin compounds were able to diffuse into the cytoplasm, it is unlikely that they will cause damage of the constituent nucleic acids. Our control compound, riboflavin also exhibits photocleavage of the plasmid (11%) which has been reported previously^45,46^. However our SIM data indicate that, due to its hydrophilic nature and lack of cationic substituent, riboflavin does not bind readily to bacterial cells resulting in limited PDI.

Collectively, this initial mechanistic study demonstrates that even in the case of increased singlet oxygen production, the key component to achieve effective Gram-negative bactericidal activity under irradiation is the presence of the guanidino group, which facilitates cell uptake and nucleic acid degradation. It should be noted, that although considered to be a powerful super resolution cross-sectional microscopy, SIM alone cannot be used as a conclusive evidence for cytoplasmic and additional strategies should be employed. However, the combination of distinct populations of bacteria in presence of different flavins in combination with enhanced DNA cleavage of guanidino compounds, speaks of at least some level of uptake.

### Photodynamic inactivation of murine hepatitis virus (MHV-A59)

Encouraged by the identification of such highly effective guanidino-flavins for bacterial inactivation, we were interested to see if the trend would be similar for coronaviruses, specifically, the coronavirus surrogate, murine hepatitis virus A59 strain (MHV-A59)^30^. It has already been demonstrated that riboflavin can effectively inactivate both enveloped and non-enveloped viruses in blood products using UV light^21–23^, but much lower efficacy was observed using visible light (0.4 × 10^5^ lx, 0.5–2 h) against hepatitis B virus (HBV)^47,48^. In order to evaluate possible applicability towards virus-inactivating surface coatings or textiles, we used an in vitro TCID_50_ assay to evaluate the viral titre of MHV-A59 through inoculation into murine fibroblast 17Cl-1 cells after irradiation at varying concentrations of flavin in PBS. The cytotoxicity of the flavins towards this cell line was first investigated by 24 h incubation MTS assay allowing us to obtain a working concentration range of ≤ 10 μM for the in vitro TCID_50_ assay to evaluate viral PDI efficacy (see ESI Figures S11 and Table S4 for 17Cl-1 cytotoxicity data).

**Figure 4:**
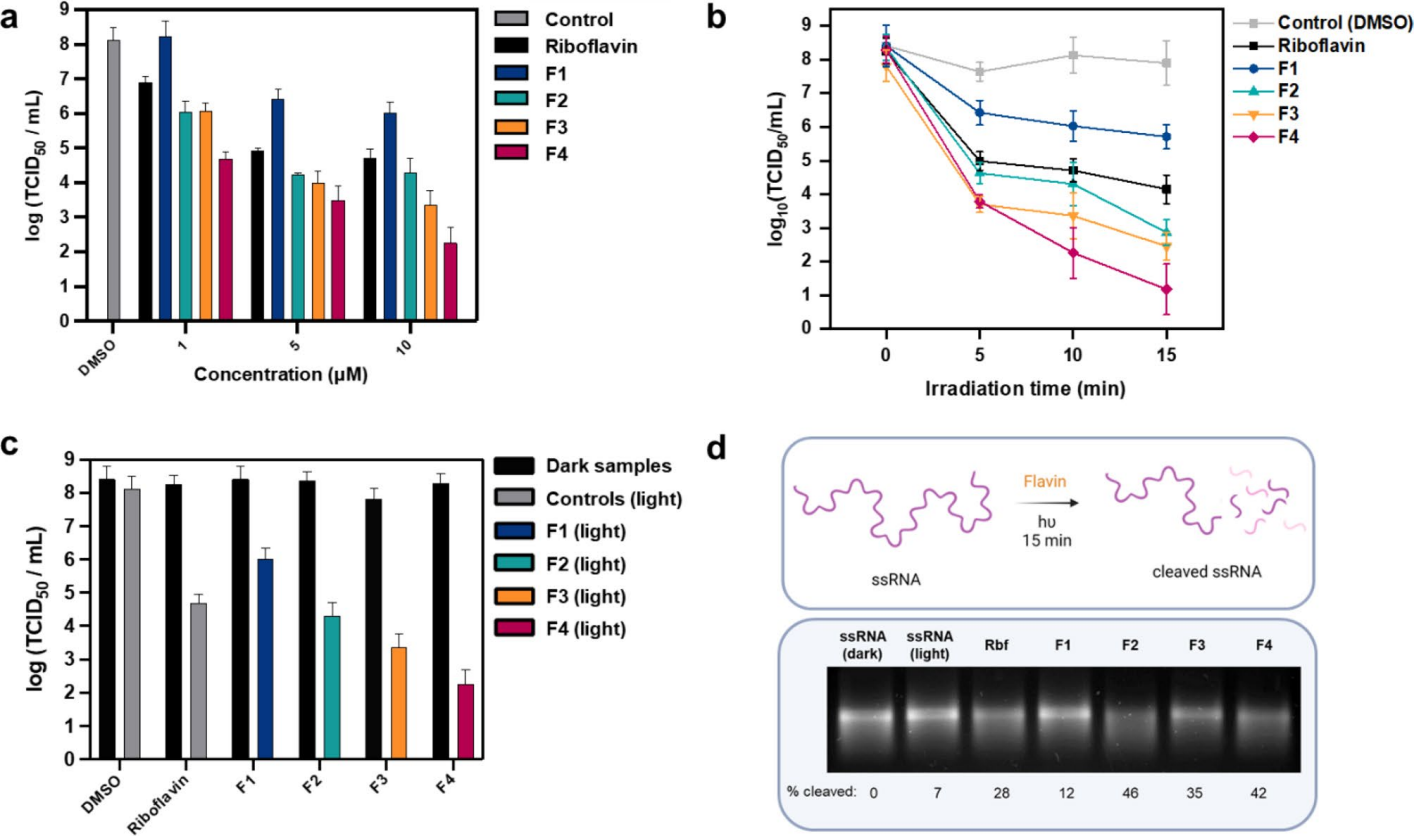
Photodynamic inactivation of murine hepatitis virus (MHV-A59). (**a**) Photodynamic inactivation of murine hepatitis virus A59 strain (MHV-A59) incubated with various concentrations of flavins irradiated with white LED light (1 × 10^5^ lx, 35 mW/cm^2^) for 10 min in PBS containing 0.1% DMSO. Data are expressed as the mean ± SD of three biological replicates. (**b**) Time course of MHV-A59 inactivation incubated with 10 µM of flavin irradiated with white LED light (1 × 10^5^ lx, 35 mW/cm^2^) for 15 min in PBS containing 0.1% DMSO. Data are expressed as the mean ± RE of three biological replicates. (**c**) Photodynamic inactivation of MHV-A59 incubated with 10 µM of flavin compound irradiated with white LED light (1 × 10^5^ lx, 35 mW/cm^2^) or incubated in the dark for 10 min in PBS containing 0.1% DMSO. Data are expressed as the mean ± SD of three biological replicates. (**d**) Agarose gel electrophoresis of ssRNA following incubation with 10 µM flavin irradiated with white LED light (1 × 10^5^ lx, 35 mW/cm^2^) for 15 min in PBS containing 0.1% DMSO. Rbf = Riboflavin. Uncropped gel electrophoresis image is shown in ESI Figure S10.

We started our investigation by varying the concentration of flavin (1–10 μM in PBS) with 10 min of white LED exposure (21.0 J/cm^2^ light dose, Fig. 4a). Even at 1 μM, the brominated guanidino flavin **F4** demonstrated a 3.4 log_10_ reduction in viral titre, while **F2** and **F3** showed 2.1 log_10_ reduction (Fig. 4a, Table S3). It should be noted that reductions of the order of 4 logs or more (> 99.99%) are considered highly effective by EMA guidance and that a > 1 log_10_ reduction in necessary to be considered reliable^49^. At the same concentration, riboflavin achieved a 1.2 log_10_ reduction in titre, however **F1** showed no effect under these conditions. Nevertheless, the activity of all flavins improved by the increase of their concentrations resulting in viral load reductions of > 99.9% in the presence of riboflavin or **F2**, and > 99.99% with **F3** or **F4** at 5 μM. Further increase in concentration (to 10 µM) only substantially improved virucidal activity for **F4** (> 5 log_10_). The irradiation time was then explored for 10 μM flavin to reveal high degrees of inactivation (≥ 5 log_10_) for **F2**, **F3** and **F4** when irradiated for 15 min (31.5 J/cm^2^ light dose) whereas shorter irradiation times (5 min, 10.5 J/cm^2^ light dose) still provided efficient inactivation of MHV-A59 ≥ 3 log_10_ steps using those same flavins (Fig. 4b). It should be noted that variability within all experiments was noticeably high which is typical for TCID_50_ assays^50^. However, no reliable log reduction of MHV-A59 was observed when irradiated without flavin in PBS containing 0.1% DMSO (Fig. 4b). To confirm that the mechanism of viral inactivation by the flavins was dependent on light, dark control experiments (10 μM, 10 min incubation) resulted in no effective virucidal activity being observed (> 1 log_10_ reduction, Fig. 4c).

The activity of the flavin derivatives towards viral inactivation show a different trend than previously observed with our model bacterium. For example, in the presence of riboflavin under the same conditions, effective PDI of MHV-A59 was measured whereas there was no activity towards *E. coli*. Therefore, it can be assumed that the requirements for a PS to interact and/or diffuse through the coronavirus membrane are less dependent on lipophilicity or the presence of cationic charge. Despite this, our flavin derivatives again show that guanidino group incorporation improves antiviral PDI activity when compared to amino groups. However, bromination now seems to play a more important role in viral deactivation as brominated amino **F3** outperformed methylated guanidino **F2**.

To try and explain our findings, we investigated the interaction of the flavins with RNA to elucidate how its light-induced cleavage could lead to coronavirus inactivation, a model ssRNA (~ 1 k nt) was irradiated (1 × 10^5^ lx) in the presence of 10 µM flavin in PBS for 15 min and the extent of cleavage was observed via agarose gel electrophoresis (Fig. 4d). It was observed that **F1** exhibits the least RNA photocleavage (12%) whereas riboflavin shows greater activity (28%) which corroborates the result of viral inactivation and confirms previous findings^51–53^. Similar to our results with pDNA, guanidino-containing **F2** and **F4** show the best photocleavage ability (46% and 42% respectively). Interestingly, **F3** also exhibits effective cleavage of RNA (35%) which was not observed in the case of pDNA (Fig. 3d). This can be rationalised by the mechanism of flavin-mediated RNA photocleavage which has been previously shown to depend more upon ^1^O_2_ oxidation than electron transfer events between flavin and nucleobase^51–53^. Taken together, it can be concluded that efficient ^1^O_2_ production is key to viral PDI using flavin derivatives.

### In vitro toxicity towards human cells

**Figure 5:**
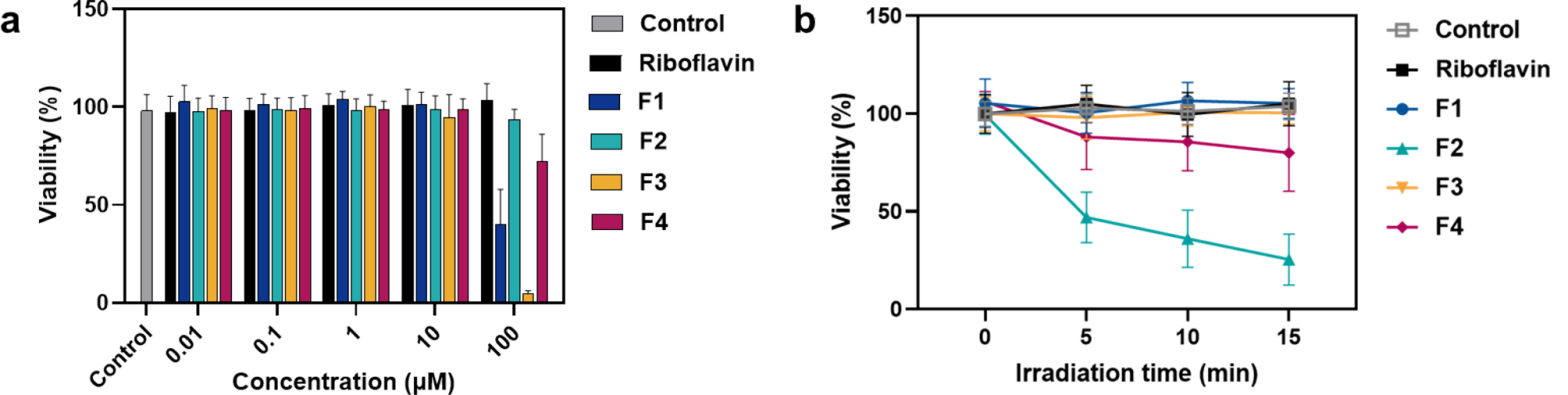
In vitro toxicity of **F1-4** towards human cells. (**a**) In vitro cytotoxicity effect of **F1–4** and riboflavin on WI-38 cells after 24 h incubation determined by MTS assay. Data are expressed as the mean ± SD of three biological replicates. (**b**) Light-induced toxicity of WI-38 cells treated with **F1**–**F4** and riboflavin (10 µM) irradiated for 0–15 min with 1 × 10^5^ lx white LED light. Data are expressed as the mean ± SD of three biological replicates. Control = 0.1% DMSO in PBS.

Finally, before considering the applications of our flavin derivatives for PDI of topical pathogen infections, surface coatings or textiles, we investigated their impact on human cells. In general, an ideal photosensitiser for these applications should show no cytotoxic effects on human cells in the dark and limited effects given the same irradiation conditions used for inactivation of the target pathogen, also referred to as a therapeutic window^12^. Accordingly, the inherent cytotoxicity of the flavins was evaluated through incubation with human lung fibroblast cells (WI-38). A 24 h MTS assay was used to calculate inhibitory concentration values (IC_50_) of > 100 µM for riboflavin and guanidino-functionalised **F2** and **F4** which can therefore be considered as non-toxic (Fig. 5a, Table S4). On the other hand, amino-functionalised **F1** and **F3** had IC_50_ values of 96.1 µM and 30.9 µM respectively (Table S4). It has been shown previously that the cytotoxicity of amino-containing compounds may be derived from an increase in intracellular amine oxidase activity that induces excess oxidative stress leading to cell apoptosis^54^.

We then studied the light-induced toxicity of the flavins towards the WI-38 fibroblast cells by observing decreases in cell viability over irradiation time using an MTS assay (Fig. 5b). Interestingly, the cytotoxicity trends observed in the dark after 24 h are reversed when exposed to light (1 × 10^5^ lx) over shorter time periods. For example, after 15 min of irradiation, 10 μM of methylated guanidino **F2** induces ~ 75% reduction in cell viability. Under the same conditions, brominated guanidino **F4** exhibits less of an effect with ~ 20% reduction in cell viability. The degree of variability in these experiments could be attributed to the amphiphilic nature of the flavins leading to aggregation of the compounds in aqueous conditions. On the other hand, riboflavin, amino-functionalised **F1** and **F3** display no decrease in cell viability over 15 min irradiation. These data show that despite being non-toxic to the cells in the dark, the guanidino-functionalised flavins can induce cytotoxicity upon irradiation most likely through similar mechanisms discussed for pathogens. It is our ongoing work to understand the differences in light induced cytotoxicity that were observed between methylated and brominated guanidino flavin derivatives. Nevertheless, **F4** could be suitable for further PDI applications thanks to effective bacterial and viral inactivation (> 3 log_10_ reduction after 15 min irradiation at 11 μM and 1 μM respectively) coupled with low toxicity to human fibroblast cells under the same conditions or in the dark.

## Conclusion

In summary, we have rationally designed small library of flavin derivatives containing functional groups that significantly improve visible light photodynamic inactivation of pathogens. By incorporating a guanidino moiety into the flavin structure (**F2** and **F4**), the inactivation efficacy against Gram-negative bacteria, *E. coli* BL21(DE3) in PBS, was remarkably enhanced compared to natural riboflavin and amino variants (**F1** and **F3**). We believe that this can be explained by enhanced cell uptake of these compounds and subsequent pDNA cleavage. Bromination of the structure (**F3** and **F4**) enhanced singlet oxygen production via the heavy-atom effect which generally led to improved PDI of *E. coli*, however the incorporation of a guanidino substituent dominated the outcome. Very effective inactivation was achieved with F4, whereby 1 min of visible irradiation (2.10 J/cm^2^ light dose) reduced the bacterial load by > 4 log_10_ steps.

It was found that the presence of guanidino group does not enhance the selectivity of the compounds in case of PDI of SARSCoV-2 surrogate, MHV-A59 when evaluating the PDI against the SARS-CoV-2 surrogate, MHV-A59 in PBS. Rather, the bigger effect is exerted by presence of bromo substituents; brominated amino **F3** had a higher effiecenty compared to methylated guanidino **F2** compound, which can be attributed to more significant ^1^O_2_-mediated RNA cleavage. This was evidenced by brominated amino **F3** having a higher efficacy compared to methylated guanidino **F2** which was attributed to greater RNA cleavage via ^1^O_2_ oxidation. The highest viral PDI was achieved by **F4** where > 4 log_10_ reduction in titre was achieved after 10 min of visible irradiation (21.0 J/cm^2^ light dose) in the presence of 5 μM compound. Toxicity studies using the novel flavins towards human fibroblast cells (WI-38) further confirmed that **F4** could be a suitable candidate for PDI applications against both bacteria and viruses.

Overall, we hope this study inspires further exploration of riboflavin’s structure to design new generations of photosensitiser compounds to treat topical bacterial infections, enabling the design of antiviral surface coatings, sprayable hybrid materials to provide the surface protection, as well as fabrics capable of efficient visible light pathogen inactivation. Such fabrics could be used to manufacture protecting suits for medical staff, as well as curtains and bedding used in intensive care units where the sterile conditions are of the outmost importance.

## Methods

### Synthesis and characterisation of flavins

Detailed experimental methods of synthesis and characterisation of flavins can be found in the Supplementary Information.

### Photodynamic inactivation of *E. coli*

An adapted method reported by Maisch et al*.* was used to evaluate the phototoxicity of flavin compounds towards *E. coli* BL21(DE3)^28^.

After an overnight aerobic culture in LB broth at 37 °C with shaking at 225 rpm, the resulting *E. coli* were centrifuged at 2,500 rpm for 10 min. The pellet was resuspended in PBS (Sigma, 1x, pH 7.4) to an OD_600_ value of 0.6 (~ 1 × 10^8^ bacteria per ml). Bacterial solutions were then combined with varying concentrations of **F1-4** and riboflavin (0, 1, 10, 100 μM) dissolved in PBS in a 1:1 ratio and left to incubate for 20 min in dark. Aliquots of negative controls (0.5% DMSO in PBS; PBS only) and 100 μM flavin-bacteria samples were covered in foil to remain in dark, while all samples were plated onto a 96-well plate and exposed to white LED irradiation (1 × 10^5^ lx) for 15 min. After 15 min of irradiation or darkness, each sample was serially diluted to its respective, appropriate dilution and plated onto an LB agar plate with 50 μg/mL kanamycin. Plates were incubated overnight at 37 °C in the dark. Survival of bacteria was determined by counting colony forming units (CFUs) the next day.

For time point data, aliquots of 100 μM flavin-bacteria solutions were taken at 5-min increments of irradiation (0, 5, 10, and 15 min) and evaluated similarly as above. Time point experiment with one-minute increments, between 0 and 5 min, were conducted for 100 μM **F4**-bacteria samples.

All data was generated with technical replicates and biological triplicates.

All statistical analysis was done with Graphpad Prism 9. One-way ANOVA with Tukey’s multiple comparisons test, was performed to evaluate the impact of flavin concentration compared to the control (PBS). Two-way ANOVA with Bonferroni’s multiple comparison test, was performed to evaluate the impact of light given the same flavin concentration with different irradiation conditions (dark and light). Significance levels are defined as the following: ns for p > 0.05, * for p ≤ 0.05, ** for p ≤ 0.01, *** for p < 0.001, and **** for p < 0.0001.

### Photodynamic inactivation of murine hepatitis virus (MHV-A59)

#### Cell culture

17Cl-1 murine fibroblasts cells and murine hepatitis virus A59 strain (MHV-A59) were kindly gifted by Professor Ian Goodfellow’s lab (Department of Pathology, University of Cambridge, UK). 17Cl-1 cells were resurrected from cold-storage cell banks and propagated using routine cell culture protocols in complete growth medium (Dulbecco’s modified Eagle’s medium low glucose 1 g/L (DMEM, Life Technologies) supplemented with 5% foetal bovine serum (Merck), 6% tryptose phosphate broth (Merck), 1 × non-essential amino acids (Gibco), 1 × antibiotic–antimycotic (Thermo Fisher Scientific) and 1 × L-Glutamine (Gibco). 17Cl-1 cells were seeded (100 μl per well) into 96-well plates (untreated, flat bottom) at 1 × 10^4^ cells/well at 37 °C for 24 h in 5% CO_2_ prior to the day of testing.

#### Virus propagation

MHV-A59 was propagated in 17Cl-1 cells at a multiplicity of infection (MOI) of 0.01 TCID_50_ per cell. MHV-A59 was clarified by centrifugation at 3000 rpm for 10 min and stored in aliquots at − 80 °C. The TCID_50_ was calculated using the Reed and Muench method^55^.

#### Flavin treatment of MHV-A59

The efficacy of the flavins were enumerated utilizing modified methods from ISO 18,184 and Leibowitz et al.^56^ Flavins **F1-4** and riboflavin, were solubilized in DMSO. 100 µM working stock solutions were then created by diluting each DMSO solution into PBS. To control for the effect of DMSO on MHV-A59, DMSO was diluted into PBS at the same concentration. A white light apparatus was set up to cover a 4 × 5 (row x column) area of a 96-well plate with the same level of light intensity (1 × 10^5^ lx). The stock solutions were then diluted in replicates of four to a total of 180 µl in PBS at 1, 5, and 10 µM concentrations for each sample. Virus stocks of MHV-A59 were thawed on ice prior to use. 20 µl of MHV-A59 stock (1.4 × 10^9^ PFU/ml) was then mixed into each well, and the 4 × 5 well matrix was treated with the light or incubated in the dark at room temperature for a specified time (5, 10, or 15 min).

#### Virus recovery and TCID_50_ Assay MHV-A59

After treatment, the samples were mixed into 320 µl of Gibco Difco™ Beef Extract (1.5% w/v beef extract in ddH_2_O; Life Technologies) and rolled for 15 min to chelate any free ions in the solution. The samples then were serially diluted (10^0^–10^–7^) in infectivity media (Dulbecco’s modified Eagle’s medium low glucose 1 g/L (DMEM, Life Technologies) supplemented with 2.5% fetal bovine serum (Merck), 3% tryptose phosphate broth (Merck), 1 × non-essential amino acids (Gibco), 1 × antibiotic–antimycotic (Thermo Fisher Scientific) and 1 × L-Glutamine (Gibco]) and added in triplicate to 17Cl-1 cell plates. For each sample or control there were 4 replicate treatments (in light or dark); each treatment replicate was plated in triplicate. Infected 17Cl-1 plates were incubated at 37 °C in a 5% CO_2_ atmosphere for 24 h. Plates were scored for cytopathic effect (CPE) by microscopy and viral titres were determined by the Reed and Muench 50% tissue culture infectious dose (TCID_50_) end point method^55^. Outliers were removed from the four replicates using an outlier test. The full protocol was repeated in triplicate on different days to account for any variability in the assay.

### Nucleic acid photocleavage

**General procedure**: Riboflavin or flavin **F1-4** (10 µM from DMSO stock solutions) were mixed with either pUC18 DNA (Thermo Scientific, 0.50 µg) or EGFP-encoding mRNA (~ 1 k nt) as the ssRNA model (TriLink Biotech, 0.88 µg) dissolved in PBS (40 µL) to give a final DMSO concentration of 0.1%. A 20 µL aliquot was taken after 15 min of white LED irradiation (1 × 10^5^ lx). Prior to loading, 2 µL of 6X Orange-G loading dye in glycerol were added to the aliquot and mixed vigorously. The gel was electrophoresed at 80 V for 35 min with 1% agarose gels in 1X Tris–acetate-EDTA (TAE) buffer. Gels were then imaged in a Syngene G:BOX Gel Documentation System and band % quantification was performed using ImageJ Gel Analyzer.

### In vitro toxicity towards human cells

#### Cell culture

Normal human lung fibroblasts, WI-38, were purchased from American Type Culture Collection (ATCC) and murine fibroblast cell line, 17Cl-1, was kindly provided by Ian Goodfellow’s lab (Department of Pathology, University of Cambridge, UK). WI-38 cells were cultured in Dubelco’s modified essential medium (Gibco) with 10% fetal bovine serum (FBS, Gibco) containing penicillin (50 IU/mL) and streptomycin (50 μg/mL) (Thermo Fisher Scientific). 17Cl-1 cells were cultured as described above. All Cell lines were maintained in a humidified environment at 37 °C with 5% CO_2_ and routinely tested to confirm the absence of Mycoplasma. All in vitro experiments were conducted between 60 and 80% confluent cultures at passage 6–15 (WI-38) and 25–35 (17Cl-1).

#### MTS viability assay

The effect on cell viability of WI-38 and 17Cl-1 cells after treatment with **F1**–**F4** and riboflavin was determined using the commercially available MTS [3-(4,5-dimethylthiazol-2-yl)-5-(3-carboxymethoxyphenyl)-2-(4-sulfophenyl)-2*H* tetrazolium] assay (Promega). The MTS tetrazolium compound is reduced by cells into a coloured formazan product which is soluble in cell culture media. It can be detected colorimetrically between 450 and 540 nm with the measured absorbance directly proportional to the amount of metabolically active cells in culture. Cells were seeded into clear 96-well plates containing 10,000 cells/well in 100 µL complete growth medium and cultured for 24 h at 37 °C and 5% CO_2_. Subsequently, cells were treated with varying concentrations of **F1**–**F4** and riboflavin (0.01–100 μM) dissolved in complete growth media containing 0.1% DMSO. After further 24 h incubation at 37 °C and 5% CO_2_, 20 µL of CelTiter 96® AQ_ueous_ One Solution (Promega) was added into each well and incubated at 37 °C, 5% CO_2_ for 1–4 h, according to the manufacturer’s instruction. The absorbance of each well was measured at 490 nm using a plate reader (Spark, Tecan). Control measurements included negative control of cells with DMEM, cells with DMEM containing 0.1% DMSO, cell-free culture media (blank) and cell-free sample dilutions in culture media to evaluate potential sample interferences with MTS assay. All experiments were conducted in biological triplicates. The percentage cell viability was calculated according to Eq. (1):

1 $$ {\text{Cell viability}} (\%) = 100 \, \times \,\left( {\frac{{{\text{Absorbance}}\, {\text{of}}\, {\text{treated}}\, {\text{cells}} - {\text{Absorbance}}\, {\text{of}}\, {\text{blank}}}}{{{\text{Absorbance}}\, {\text{of}}\, {\text{control}} - {\text{Absorbance}}\, {\text{of}}\, {\text{blank}}}}} \right) $$

An adapted method reported by Maisch et al*.* was used to conduct phototoxicity studies on human lung fibroblasts, WI-38^28^. Cells were seeded into 96-well plates containing 10,000 cells/well in 100 µL complete growth medium and cultured for 24 h at 37 °C and 5% CO_2_. On the next day, the growth media was removed, and cells were treated with 10 μM **F1**–**F4** and riboflavin dissolved in DMEM without serum and phenol red (Gibco) containing 0.1% DMSO. The resulting mixtures were then incubated in the dark for 5 min and then either illuminated with 1 × 10^5^ lx for 5, 10 or 15 min, or incubated further in the dark for 15 min (dark control). After irradiation, the flavin solutions were removed and 100 μL of DMEM with 10% FBS and without phenol red was added to each well and incubated over night at 37 °C and 5% CO_2_. Subsequently, 20 µL of MTS reagent was added into each well and incubated at 37 °C and 5% CO_2_ for 1–4 h and the absorbance of each well was measured at 490 nm using a plate reader. Control measurements included negative control untreated cells with light and in the dark containing 0.1% DMSO and cells treated with flavins for 15 min in the dark. All experiments were conducted in biological triplicates. The percentage cell viability was calculated according to Eq. (1).

## Supplementary Information

Supplementary Information.

## Data Availability

The data that support the findings of this study are available from the corresponding author upon reasonable request.
