# Supplementary material for: Tuning riboflavin derivatives for photodynamic inactivation of pathogens
Source: Sci Rep. 2022 Apr 21;12:6580. doi: 10.1038/s41598-022-10394-7 (PMC9022420; doi:10.1038/s41598-022-10394-7)
Supplement: Supplementary file 1 — Supplementary Information. [file 41598_2022_10394_MOESM1_ESM.pdf]

Electronic Supplementary Information

Tuning Riboflavin Derivatives for Photodynamic Inactivation of Pathogens

Leander B. Crocker, Ju-Hyun Lee, Suraj Mital, Gabrielle C. Mills, Sina Schack, Andrea Bistrovic-Popov, Christoph O. Franck, Ioanna Mela, Clemens F. Kaminski, Graham Christie, Ljiljana Fruk*

* Department of Chemical Engineering and Biotechnology, University of Cambridge, Philippa Fawcett Drive, Cambridge, CB3 0AS, UK

E-mail: lf389@cam.ac.uk

1 Table of Contents

1 Table of Contents 2
2 Light Source 3
3 Synthesis and Characterisation of Flavins 4
3.1 Materials and Methods 4
3.2 Characterisation techniques 4
3.3 Flavins F1 and F2 5
3.4 Flavins F3 and F4 14
4 Fluorescence Quantum Yield Calculations 27
5 Singlet Oxygen Quantum Yield Calculations 28
6 Flavin Photostability 30
7 Bacterial Culture and Phototoxicity Assay 31
7.1 E. coli Log_{10} Reductions 32
7.2 SIM Imaging 33
8 Virology and $TCID_{50}$ Assay 38
8.1 MHV-A59 Log_{10} Reductions 38
9 Viability and Phototoxicity Assay 40
10 References 42

2 Light Source

The light source used for experiments was an EvoluChem 6200K white LED (18W, 400-700 nm, λ_{max} = 445 nm, Hepatochem, US) with emission spectrum as shown in **Figure S1**. Illuminance was measured using an RS PRO IM720 Light Meter. Irradiance of emission was measured using a Thor Labs PM100D with a S120VC sensor.

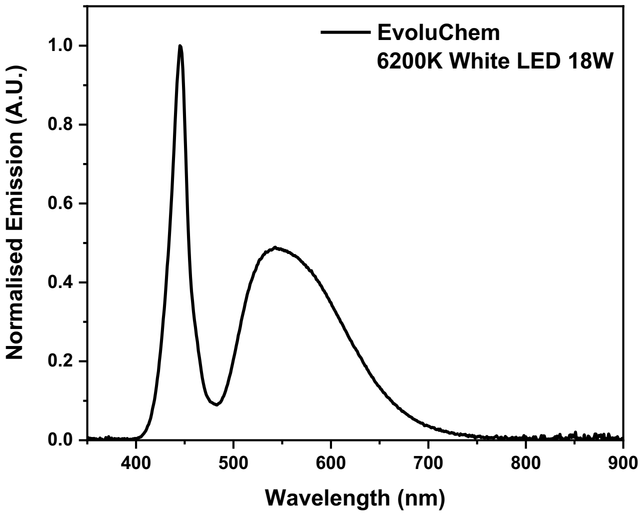

Figure S1: Emission spectrum of EvoluChem 6200K white LED light source.

3 Synthesis and Characterisation of Flavins

3.1 Materials and Methods

All materials were purchased from either Fisher Scientific (UK), Acros Organics (UK), Alfa Aeser (UK), Sigma-Aldrich (UK) or TCI Chemicals (BE) in the highest purity available and used without further purification. For column chromatography 40-63 µm silica gel (Merck) was used as stationary phase. Analytical thin layer chromatography (TLC) was performed on aluminum foil pre-coated with SiO_2-60 F254 (Merck) and visualized with a UV-lamp (254 and 365 nm).

3.2 Characterisation techniques

^{1}H and ^{13}C NMR measurements were carried out using a 400 MHz QNP Cryoprobe Spectrometer (Bruker) by the NMR service of the Department of Chemistry, University of Cambridge. The resonance multiplicity is abbreviated as: s (singlet), d (doublet), t (triplet), q (quadruplet), quint (quintet), sext (sextet), sep (septet), m (multiplet) and br (broad). The chemical shift δ is expressed in "ppm" and the coupling constant J is in "Hz". The chemical shifts are referenced to the residual solvent peak as the internal standard. HRMS was recorded on a ThermoFinnigan Orbitrap Classic (Fisher Scientific) by the MS service of the Department of Chemistry, University of Cambridge. UV-Vis absorption spectra were obtained with an Agilent Cary 300 Spectrophotometer. Fluorescence emission spectra were obtained using a Varian Cary Eclipse Fluorescence Spectrophotometer using excitation and emission slits of 5 nm.

3.3 *Flavins F1 and F2*

Flavin **1** was prepared as previously reported by Nehme *et al.*[1]

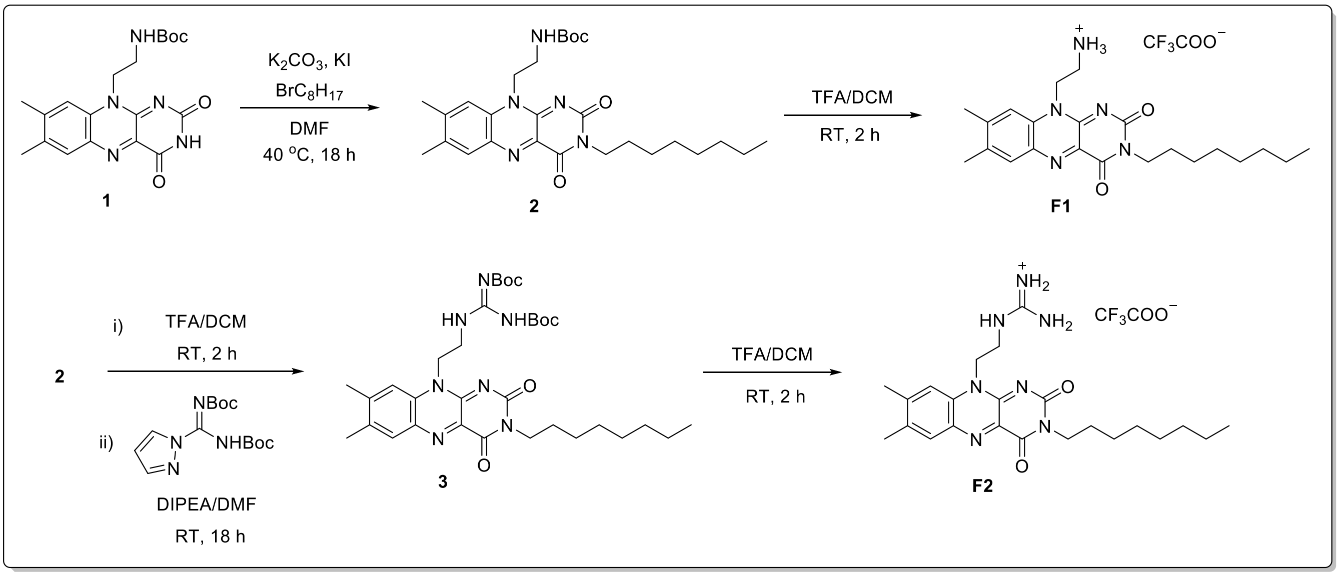

Scheme S1: Synthesis of dimethyl flavins **F1** and **F2.**

***tert*-Butyl (2-(7,8-dimethyl-3-octyl-2,4-dioxo-3,4-dihydrobenzo[*g*]pteridin-10(2*H*)-yl)ethyl)carbamate (2):**

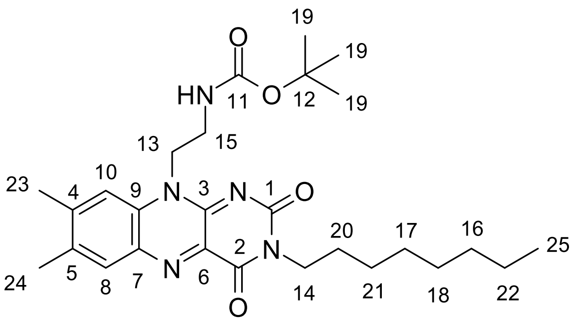

Flavin **1** (0.50 g, 1.30 mmol, 1.0 equiv.)[1], anhydrous K_2CO_3 (0.27 g, 1.95 mmol, 1.5 equiv.) and anhydrous KI (21.5 mg, 0.130 mmol, 0.1 equiv.) were suspended in anhydrous DMF (50 mL) under Ar atmosphere. The mixture was then heated to 40 °C and 1-bromooctane (1.26 g, 1.12 mL, 6.50 mmol, 5.0 equiv.) dissolved in anhydrous DMF (5 mL) was added dropwise. The mixture was then stirred for 18 h at 40 °C under Ar atmosphere in the dark. The solvent was then removed under reduced pressure and the resulting residue was suspended in EtOAc (20 mL) and washed with water (3 x 20 mL) and brine (20 mL). The organic phase was then dried over Na_2SO_4, filtered, and concentrated under reduced pressure. The resulting orange residue was purified by silica gel column chromatography (1:2 EtOAc/DCM to 1:1 EtOAc/DCM) to yield flavin **2** as an orange solid (0.544 g, 70%).

^{1}H NMR (400 MHz, $CDCl_3$) δ = 7.96 (s, 1H, **H8**), 7.84 (s, 1H, **H10**), 5.16 (br t, 1H, -N**H**-), 4.81 (t, *J* = 6.7 Hz, 2H, **H14**), 4.03 (t, *J* = 7.6 Hz, 2H, **H13**), 3.62 (br m, 2H, **H15**), 2.53 (s, 3H, **H23**), 2.41 (s, 3H, **H24**), 1.67 (quint, *J* = 7.4 Hz, 2H, **H20**), 1.39 (s, 9H, **H19**), 1.36-1.22 (m, 10H, **H16/17/18/21/22**), 0.84 (t, *J* = 6.9 Hz, 3H, **H25**) ppm.

^{13}C NMR (100 MHz, $CDCl_3$) δ = 159.7 (**C1**), 156.5 (**C11**), 155.6 (**C2**), 148.7 (**C3**), 148.1 (**C4**), 136.7 (**C5**), 135.4 (**C6**), 134.9 (**C7**), 132.3 (**C8**), 131.5 (**C9**), 115.8 (**C10**), 80.0 (**C12**), 44.1 (**C13**), 42.1 (**C14**), 37.8 (**C15**), 31.8 (**C16**), 29.3 (**C17**), 29.2 (**C18**), 28.3 (**C19**), 27.8 (**C20**), 27.0 (**C21**), 22.6 (**C22**), 21.7 (**C23**), 19.5 (**C24**), 14.1 (**C25**) ppm.

HRMS (ESI) m/z: $[M + H]^+$ Calcd for $C_{27}H_{40}O_4N_5$ 498.3080; Found 498.3074.

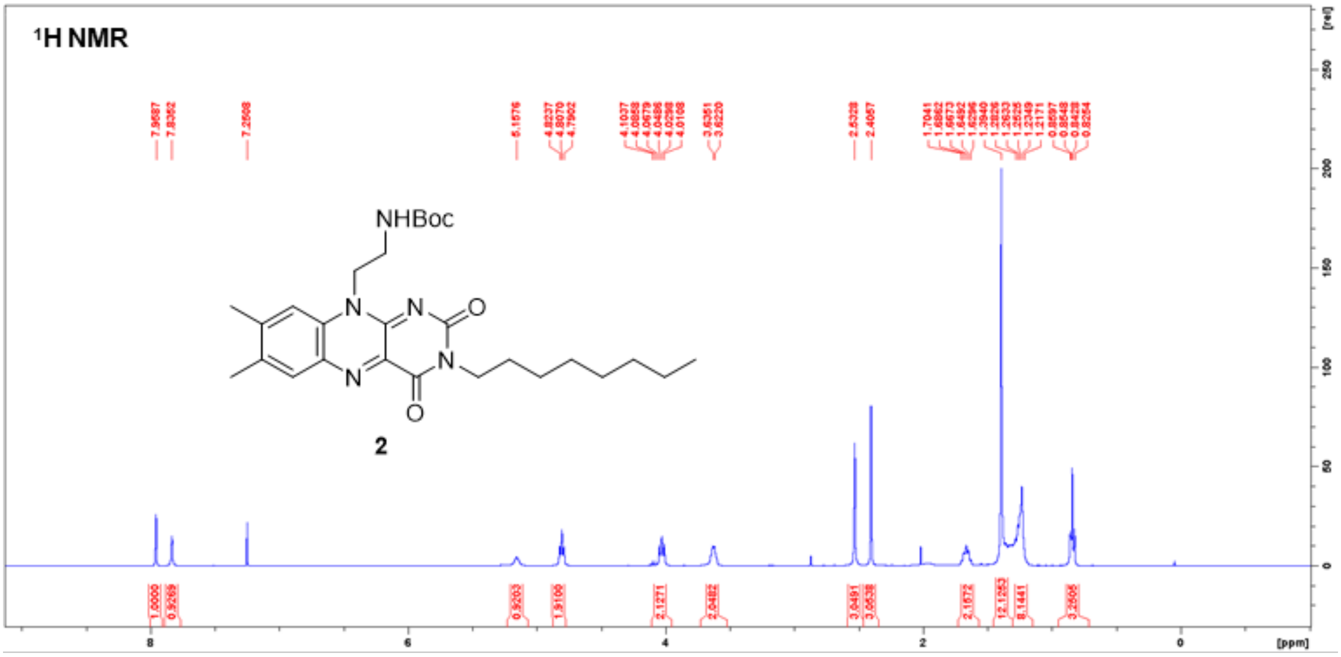

1H NMR
NHBoc
2
[ppm]
[rel]

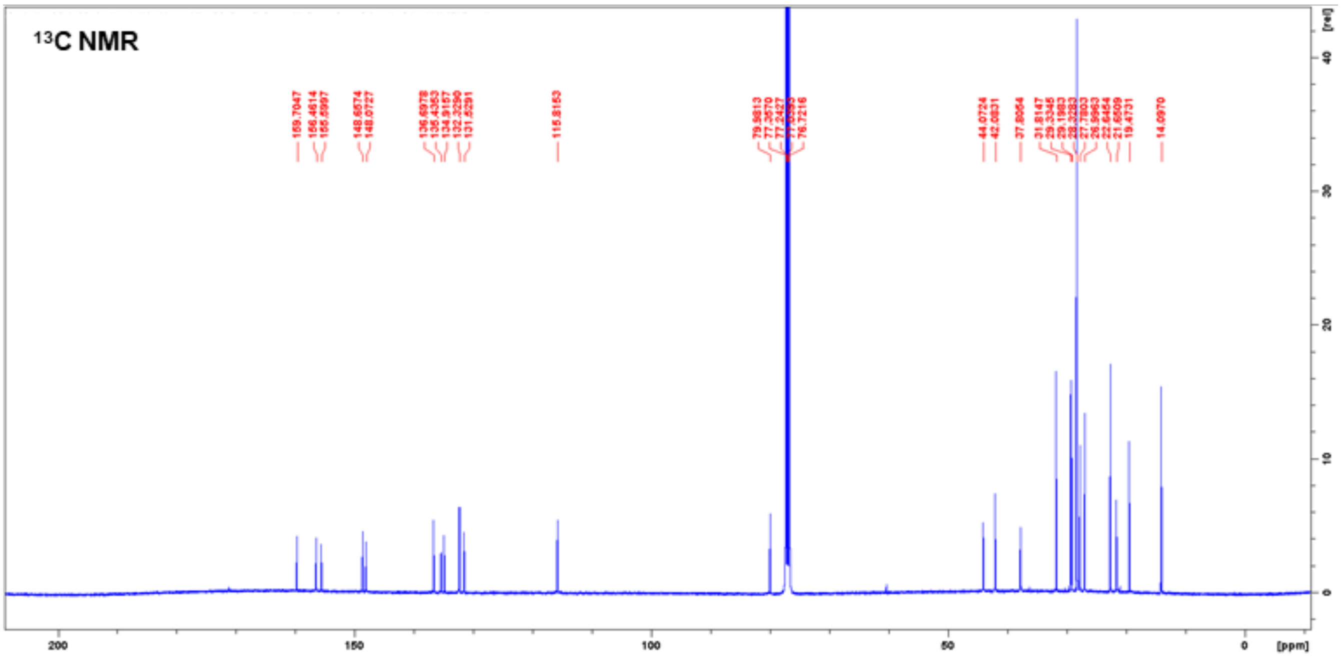

13C NMR
[ppm]
[rel]

2-(7,8-Dimethyl-3-octyl-2,4-dioxo-3,4-dihydrobenzo[g]pteridin-10(2H)-yl)ethan-1-aminium trifluoroacetate salt (F1):

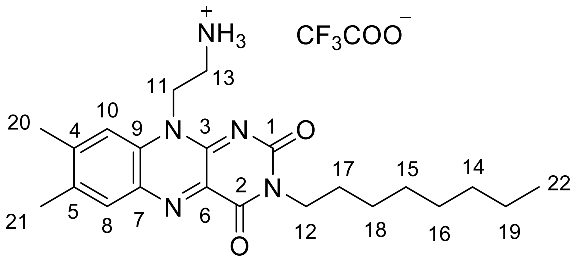

Flavin **2** (244 mg, 0.49 mmol) was dissolved in DCM (5 mL) and TFA (2 mL) was added dropwise. The resulting mixture was stirred at RT for 2 h (reaction completion judged by TLC) before the solvent was removed under reduced pressure. Excess TFA was removed using toluene (20 mL) and DCM (3 x 20 mL) co-evaporation to yield the TFA salt of flavin **F1** as a yellow solid (248 mg, 99%).

^{1}H NMR (400 MHz, DMSO-d$_6$) δ = 8.01 (br s, 2H, **-NH$_2$**), 7.95 (s, 1H, **H10**), 7.82 (s, 1H, **H8**), 4.87 (t, *J* = 6.3 Hz, 2H, **H12**), 3.87 (t, *J* = 7.4 Hz, 2H, **H11**), 3.23 (br m, 2H, **H13**), 2.50 (s, 3H, **H20**), 2.40 (s, 3H, **H21**), 1.56 (quint, *J* = 7.3 Hz, 2H, **H17**), 1.32-1.23 (m, 10H, **H14/15/16/18/19**), 0.85 (t, *J* = 6.8 Hz, 3H, **H22**) ppm.

^{13}C NMR (100 MHz, DMSO-d$_6$) δ = 159.4 (**C1**), 154.9 (**C2**), 149.8 (**C3**), 147.2 (**C4**), 136.5 (**C5**), 136.3 (**C6**), 134.3 (**C7**), 131.4 (**C8**), 130.6 (**C9**), 115.8 (**C10**), 41.2 (**C11**), 41.0 (**C12**), 36.7 (**C13**), 31.4 (**C14**), 28.9 (**C15**), 28.8 (**C16**), 27.5 (**C17**), 26.6 (**C18**), 22.2 (**C19**), 20.7 (**C20**), 18.9 (**C21**), 14.1 (**C22**) ppm.

HRMS (ESI) m/z: $[M + H]^+$ Calcd for $C_{22}H_{32}O_2N_5$ 398.2556; Found 398.2564.

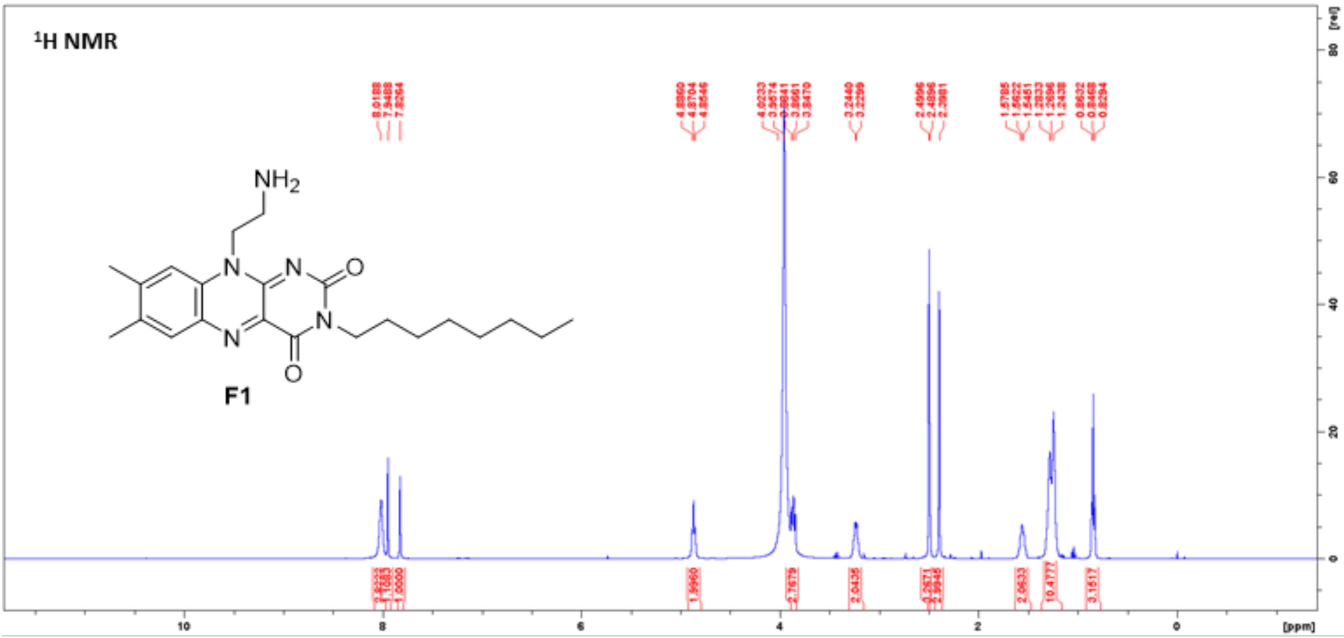
1H NMR
NH2
F1
[ppm]
[rel]

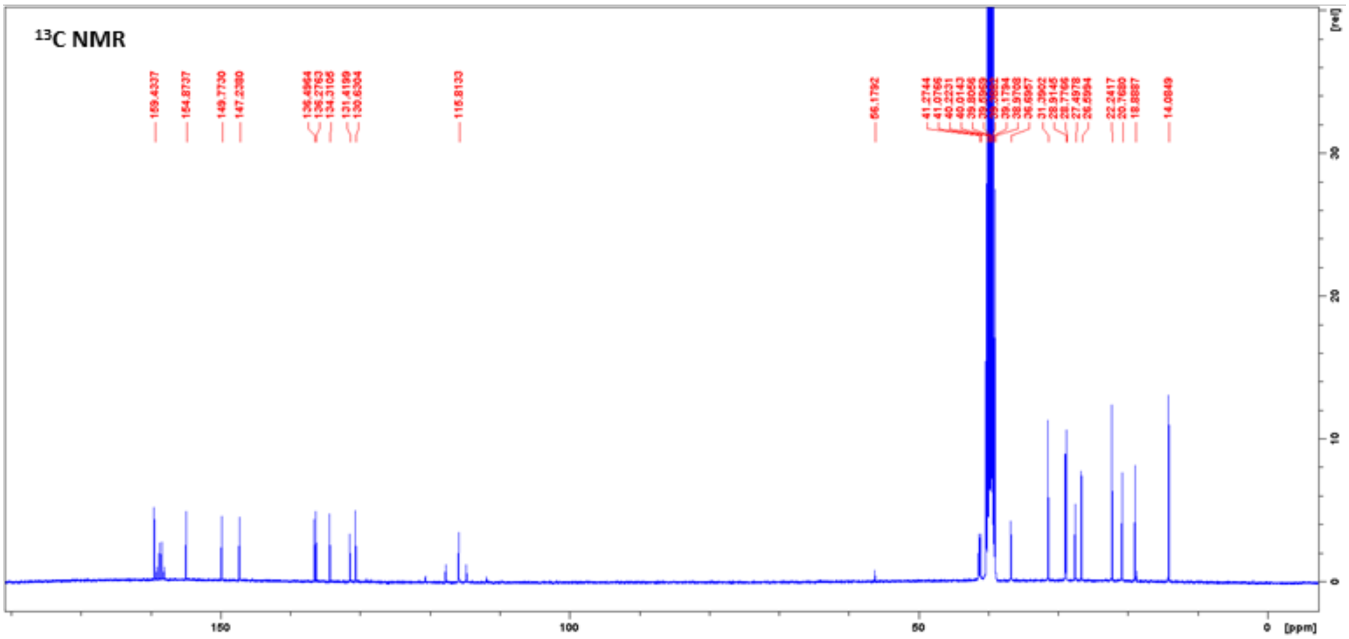
13C NMR
[ppm]
[rel]

1,3-Di-Boc-2-(2-(7,8-dimethyl-3-octyl-2,4-dioxo-3,4-dihydrobenzo[g]pteridin-10(2H)-yl)ethyl)guanidine (3):

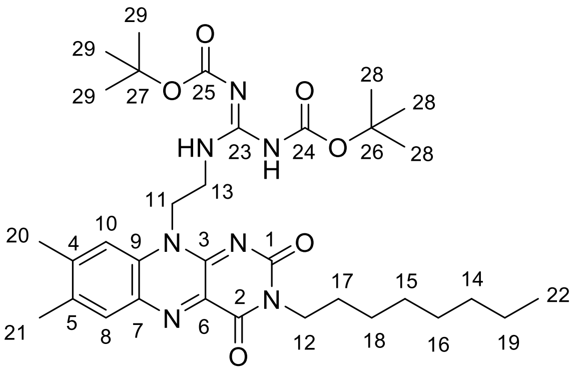

Flavin **2** (0.244 g, 0.49 mmol) was dissolved in DCM (5 mL) and TFA (2 mL) was added dropwise. The resulting mixture was stirred at RT for 2 h (reaction completion judged by TLC) before the solvent was removed under reduced pressure. Excess TFA was removed using toluene (3 x 20 mL) and DCM (3 x 20 mL) co-evaporation to yield flavin **1** which was then dissolved in anhydrous DMF (5 mL) under Ar atmosphere. DIPEA (0.17 mL, 0.98 mmol, 2.0 equiv.) was then added, followed by *N*,*N*′-di-Boc-1*H*-pyrazole-1-carboxamidine (152 mg, 0.49 mmol, 1.0 equiv). The mixture was then stirred for 18 h at RT under Ar atmosphere in the dark. The solvent was then removed under reduced pressure and the resulting residue was suspended in EtOAc (20 mL) and washed with water (3 x 20 mL) and brine (20 mL). The organic phase was then dried over Na_2SO_4, filtered, and concentrated under reduced pressure. The resulting yellow residue was purified by silica gel column chromatography (10% EtOAc/DCM) to yield flavin **3** as a yellow solid (282 mg, 90% over two steps).

^{1}H NMR (400 MHz, $CDCl_3$) δ = 11.42 (br s, 1H, -**NH**-), 8.72 (br s, 1H, -**NH**-), 8.00 (s, 1H, **H10**), 7.25 (s, 1H, **H8**), 4.93 (t, *J* = 7.0 Hz, 2H, **H12**), 4.07 (t, *J* = 7.5 Hz, 2H, **H11**), 3.80 (br m, 2H, **H13**), 2.58 (s, 3H, **H20**), 2.41 (s, 3H, **H21**), 1.70 (quint, *J* = 7.42 Hz, 2H, **H17**), 1.51 (s, 9H, **H28**), 1.45 (s, 9H, **H29**), 1.40-1.19 (m, 10H, **H14/15/16/18/19**), 0.85 (t, *J* = 6.9 Hz, 3H, **H22**) ppm.

^{13}C NMR (100 MHz, $CDCl_3$) δ = 163.4 (**C23**), 159.8 (**C1**), 156.9 (**C24**), 155.7 (**C2**), 152.8 (**C25**), 148.7 (**C3**), 148.2 (**C4**), 136.6 (**C5**), 135.6 (**C6**), 134.8 (**C7**), 132.4 (**C8**), 131.3 (**C9**), 116.1 (**C10**), 83.7 (**C26**), 79.9 (**C27**), 42.2 (**C11**), 42.1 (**C12**), 37.2 (**C13**), 31.8 (**C14**), 29.4 (**C15**), 29.2 (**C16**), 28.4 (**C28**), 28.0 (**C29**), 27.8 (**C17**), 27.0 (**C18**), 22.7 (**C19**), 21.9 (**C20**), 19.5 (**C21**), 14.1 (**C22**) ppm.

HRMS (ESI) m/z: $[M + H]^+$ Calcd for $C_{33}H_{50}O_6N_7$ 640.3823; Found 640.3824.

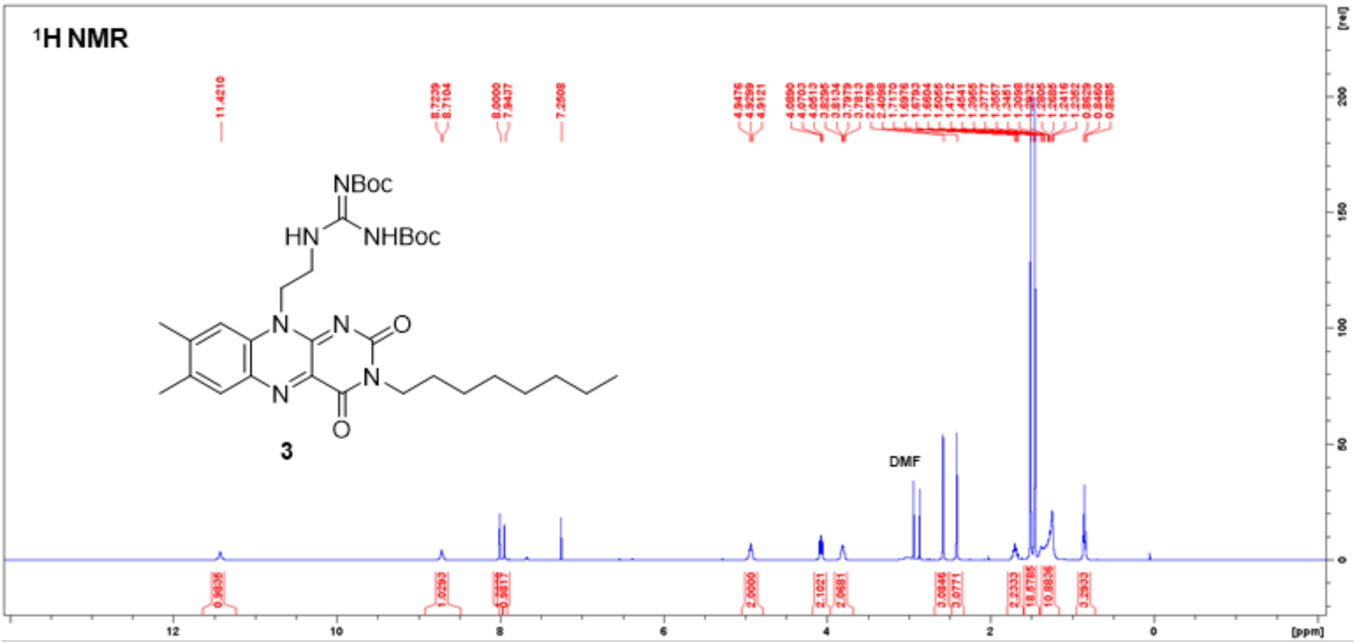
^{1}H NMR
NBoc
HN
NHBoc
N
O
3
DMF
[ppm]
[rel]

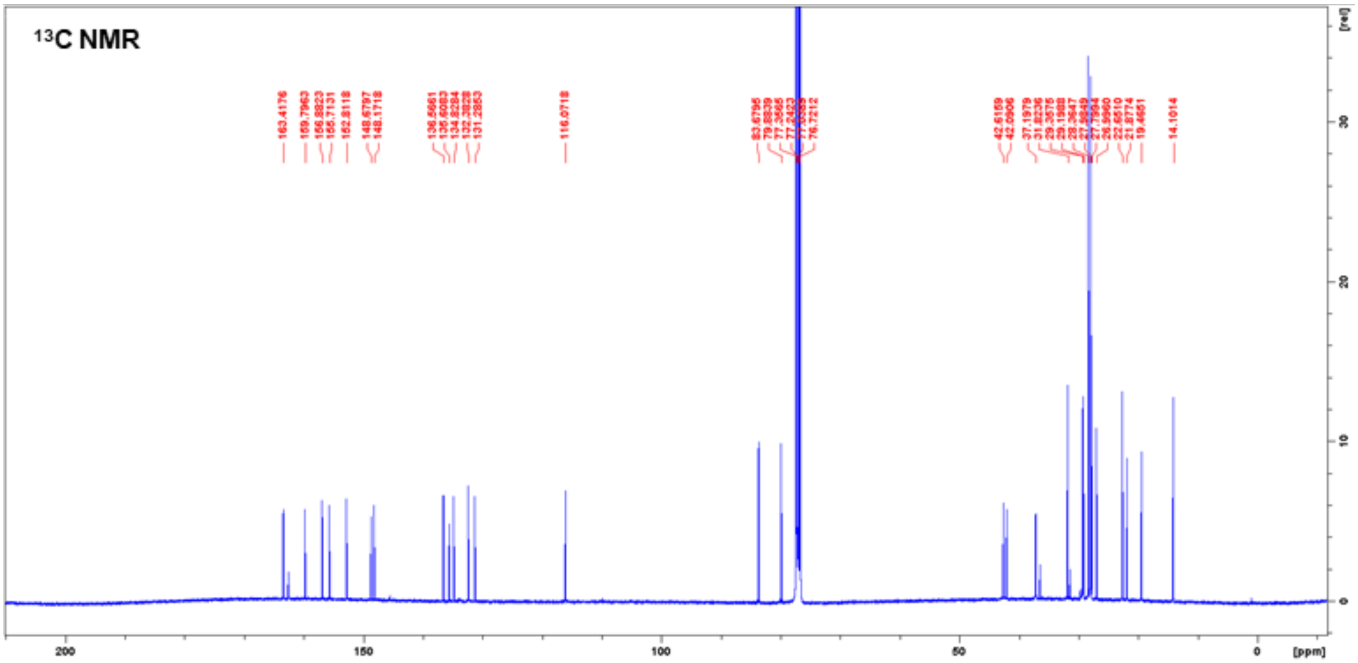
^{13}C NMR
[ppm]
[rel]

1-(2-(7,8-Dimethyl-3-octyl-2,4-dioxo-3,4-dihydrobenzo[g]pteridin-10(2H)-yl)ethyl)guanidinium trifluoroacetate salt (F2):

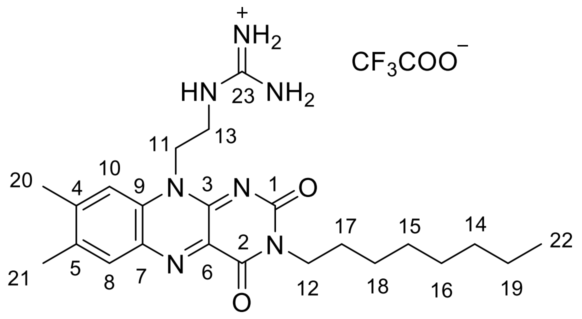

Flavin **3** (272 mg, 0.43 mmol) was dissolved in DCM (4 mL) and TFA (1.5 mL) was added dropwise. The resulting mixture was stirred at RT for 2 h (reaction completion judged by TLC) before the solvent was removed under reduced pressure. Excess TFA was removed using toluene (20 mL) and DCM (3 x 20 mL) co-evaporation to yield the TFA salt of flavin **F2** as a yellow solid (235 mg, 99%).

^{1}H NMR (400 MHz, DMSO-d$_6$) δ = 7.96 (s, 1H, **H8**), 7.81 (s, 1H, **H10**), 7.61 (br t, *J* = 6.2 Hz, 1H, -**NH**-) 4.71 (t, *J* = 6.3 Hz, 2H, **H12**), 4.48 (br s, 3H, =**NH**, -**NH$_2$**), 3.88 (t, *J* = 7.4 Hz, 2H, **H11**), 3.59 (br m, 2H, **H13**), 2.50 (s, 3H, **H20**), 2.41 (s, 3H, **H21**), 1.58 (br m, *J* = 7.42 Hz, 2H, **H17**), 1.33-1.23 (m, 10H, **H14/15/16/18/19**), 0.86 (t, *J* = 6.8 Hz, 3H, **H22**) ppm.

^{13}C NMR (100 MHz, DMSO-d$_6$) δ = 159.4 (**C1**), 157.4 (**C23**), 154.7 (**C2**), 148.9 (**C3**), 147.0 (**C4**), 136.2 (**C5, C6**), 134.3 (**C7**), 131.2 (**C8**), 130.9 (**C9**), 115.9 (**C10**), 43.0 (**C11**), 41.0 (**C12**), 37.6 (**C13**), 31.3 (**C14**), 28.9 (**C15**), 28.7 (**C16**), 27.4 (**C17**), 26.5 (**C18**), 22.2 (**C19**), 20.7 (**C20**), 18.9 (**C21**), 14.0 (**C22**) ppm.

HRMS (ESI) m/z: $[M + H]^+$ Calcd for $C_{23}H_{34}O_2N_7$ 440.2774; Found 440.2783.

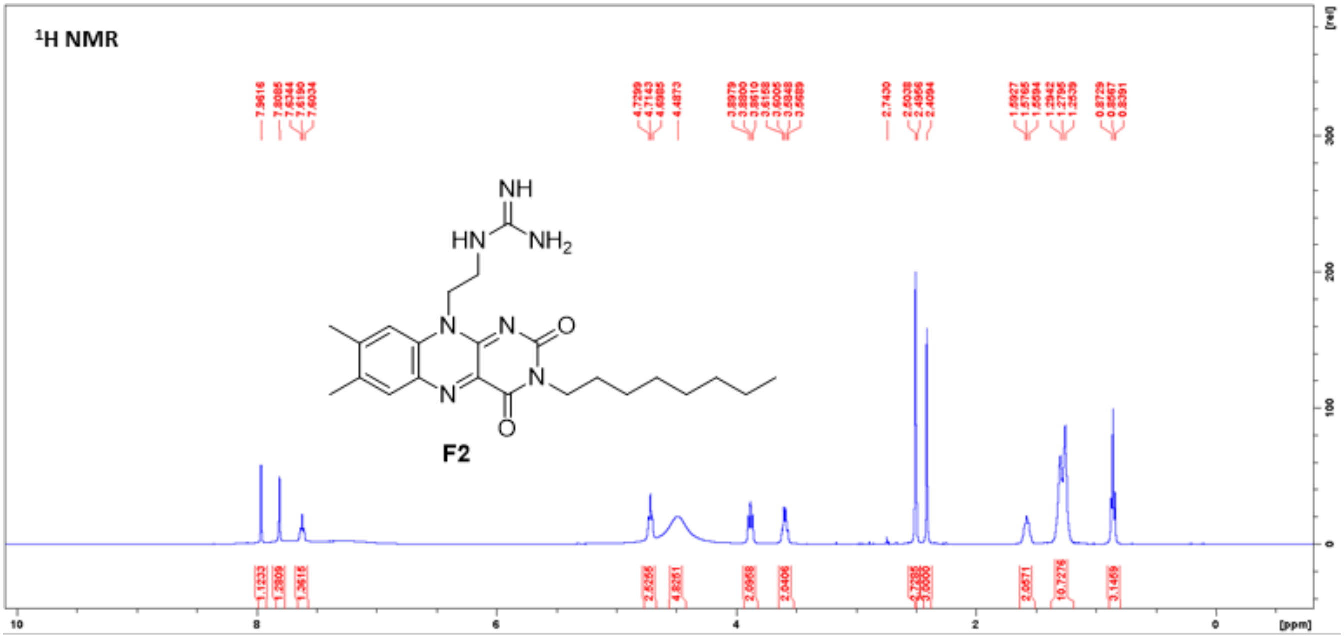
^{1}H NMR
F2

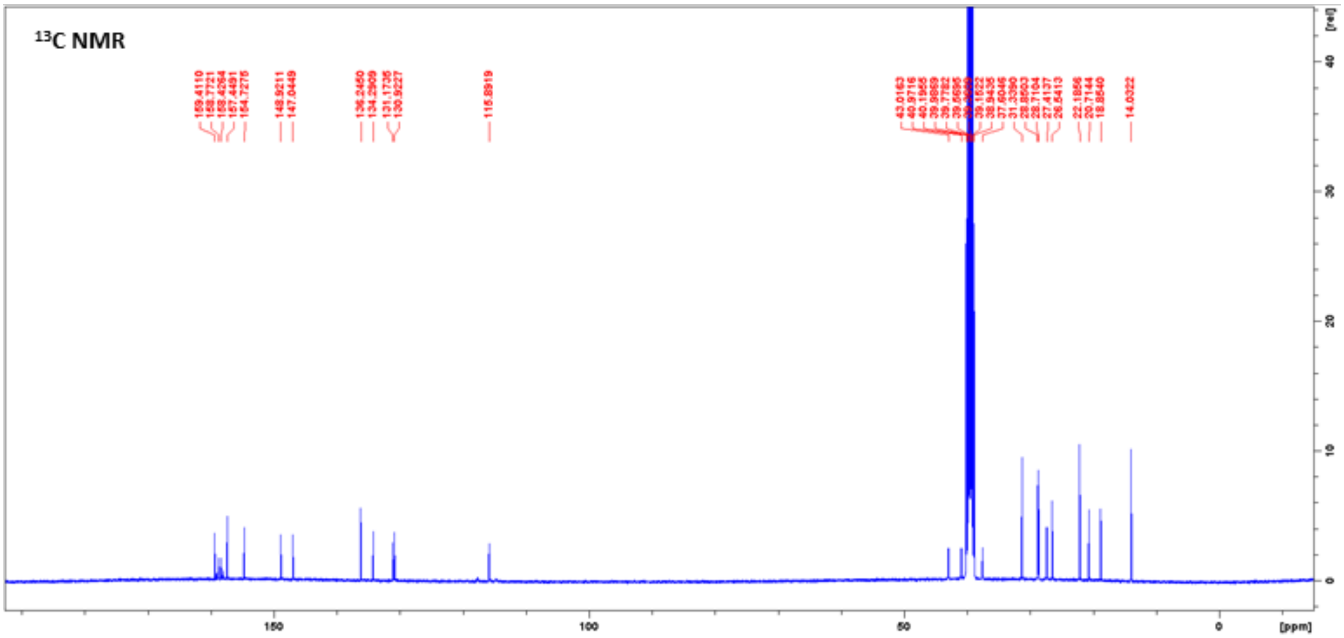
^{13}C NMR

3.4 Flavins F3 and F4

1,2,4-Tribromo-5-nitrobenzene (**4**) was prepared as previously reported by Legrand *et al.*[2]

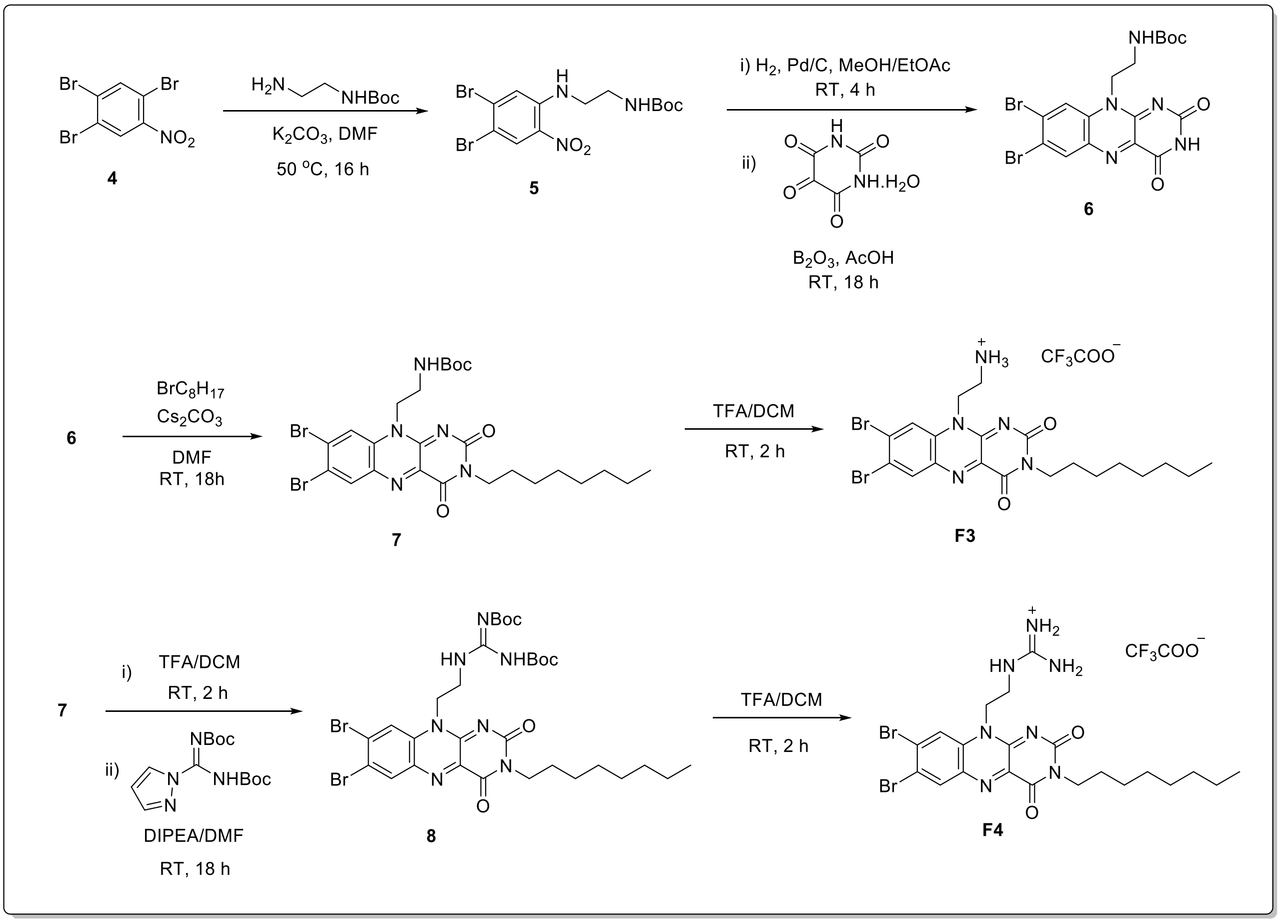

Scheme S2: Synthesis of flavins **F3** and **F4.**

***tert*-Butyl (2-((4,5-dibromo-2-nitrophenyl)amino)ethyl)carbamate (5):**

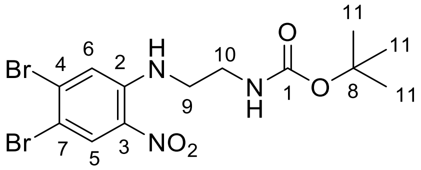

1,2,4-Tribromo-5-nitrobenzene (**4**) (1.00 g, 2.78 mmol, 1.0 equiv.) and anhydrous K_2CO_3 (576 mg, 4.17 mmol, 1.5 equiv.) were suspended in anhydrous DMF (25 mL) before adding a solution of *tert*-butyl (2-aminoethyl)carbamate (1.70 g, 10.61 mmol, 5.0 equiv.) in anhydrous DMF (5 mL) dropwise at RT under Ar atmosphere. The resulting mixture was then stirred at 50 ^oC for 18 h. The solvent was then removed under reduced pressure and the resulting residue was suspended in EtOAc (20 mL) and washed with water (3 x 20 mL) and brine (20 mL). The organic phase was then dried over Na_2SO_4, filtered, and concentrated under reduced pressure. The resulting residue was purified by silica gel column chromatography (5-10% EtOAc/Cyclohexane) to yield **5** as an orange solid (785 mg, 64%).

^{1}H NMR (400 MHz, $CDCl_3$) δ = 8.37 (s, 1H, **H5**), 8.08 (br s, 1H, -**NH**-), 7.21 (s, 1H, H6), 4.83 (br s, 1H, Ar-**NH**-), 3.45-3.40 (br m, 4H, **H9**, **H10**), 1.45 (s, 9H, **H11**) ppm.
^{13}C NMR (100 MHz, $CDCl_3$) δ = 156.2 (**C1**), 144.3 (**C23**), 134.1 (**C2**), 131.7 (**C3**), 130.6 (**C4**), 118.3 (**C5**), 109.4 (**C6**), 80.1 (**C7**), 43.5 (**C8**), 39.5 (**C9**), 28.3 (**C10**) ppm.

HRMS (ESI) m/z: $[M + H]^+$ Calcd for $C_{13}H_{17}O_4N_3Br_2Na$ 459.9484; Found 459.9470.

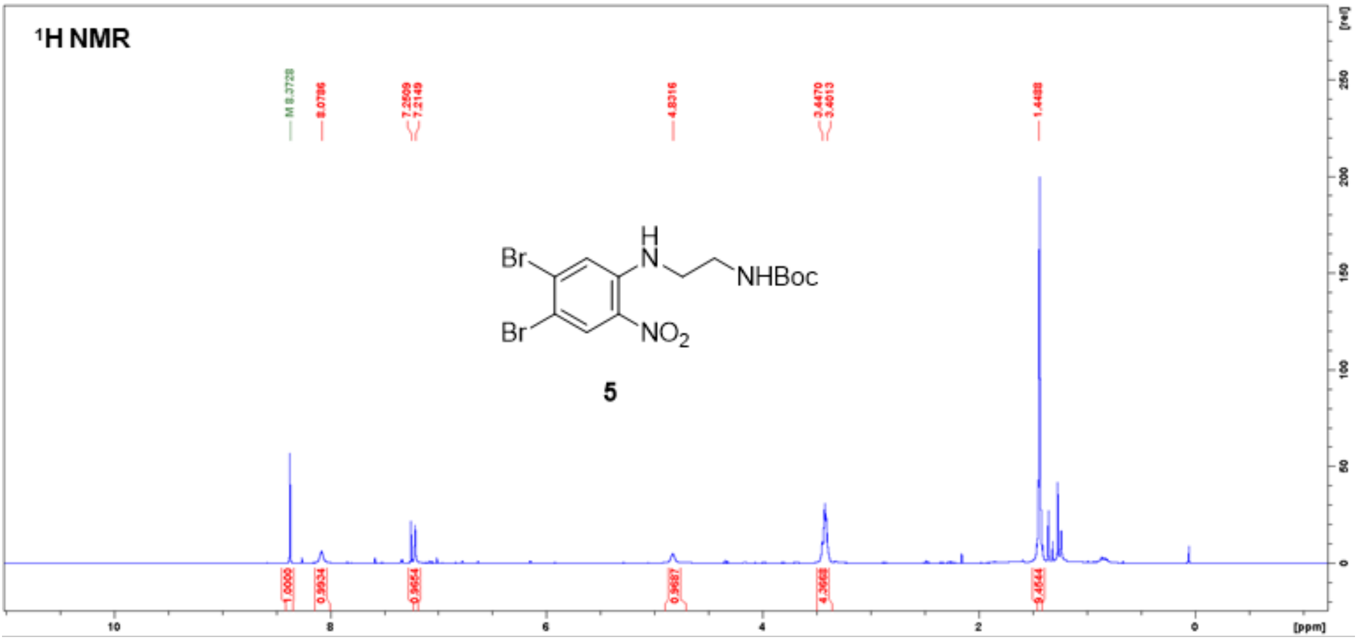
1H NMR
M 8.3728
8.0786
7.2509
7.2149
4.8316
3.4470
3.4013
1.4488
Br
Br
H
N
NHBoc
NO2
5
1.0000
0.9934
0.9654
0.9687
4.3668
9.4544
[ppm]
[rel]

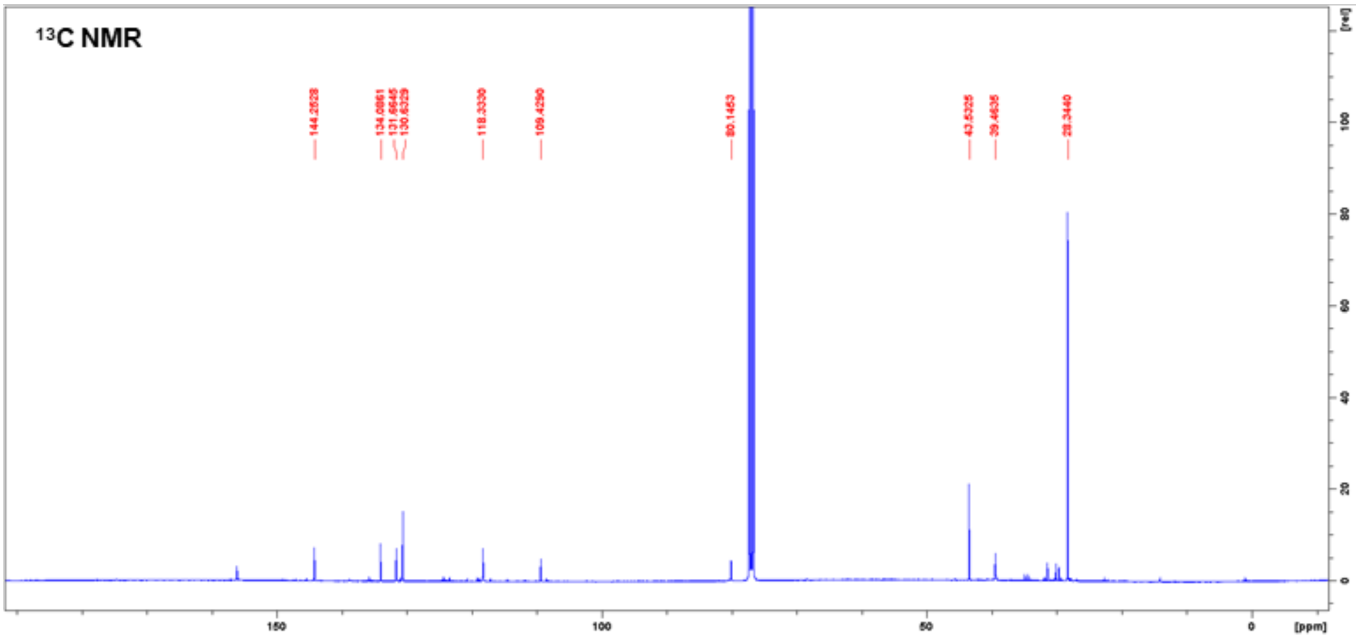
13C NMR
144.2528
134.0861
131.6645
130.6329
118.3330
109.4290
80.1453
43.5325
39.4635
28.3440
[ppm]
[rel]

***tert*-Butyl (2-(7,8-dibromo-2,4-dioxo-3,4-dihydrobenzo[*g*]pteridin-10(2*H*)-yl)ethyl)carbamate (6):**

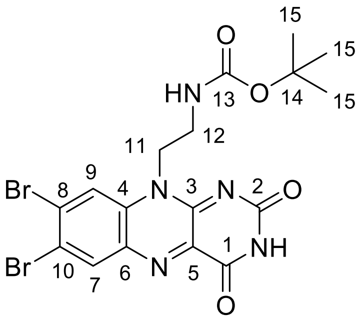

tert-Butyl (2-((4,5-dibromo-2-nitrophenyl)amino)ethyl)carbamate (**5**) (785 mg, 1.78 mmol, 1.0 equiv.) was dissolved in MeOH/EtOAc (1:1 v/v, 20 mL) and system purged with Ar. Pd/C (50 mg) was then added and the atmosphere was replaced with H_2 gas (2 x balloons). The resulting mixture was stirred for 4 h (reaction completion judged by TLC) at RT before being filtered through Celite and concentrated under reduced pressure. The resulting red residue was then dissolved in glacial acetic acid (20 mL) under Ar atmosphere. Subsequently, B_2O_3 (248 mg, 3.56 mmol, 2.0 equiv.) was added, followed by alloxan monohydrate (285 mg, 1.78 mmol, 1.0 equiv) and the resulting reaction mixture was stirred for 18 h under Ar atmosphere in the dark. The mixture was then partitioned between EtOAc (20 mL) and water (20 mL), and the organic phase was washed with brine (3 x 20 mL), dried over Na_2SO_4, filtered and concentrated under reduced pressure. The orange residue was then purified by silica gel column chromatography (1% EtOH/EtOAc) to yield flavin **6** as an orange solid (313 mg, 34% over two steps).

^{1}H NMR (400 MHz, DMSO-d_6) δ = 11.57 (s, 1H, Ar-**NH**), 8.52 (s, 1H, **H7**), 8.44 (s, 1H, **H9**), 7.00 (br t, *J* = 6.0 Hz, 1H, -**NH**-), 4.59 (t, *J* = 5.2 Hz, 2H, **H11**), 3.36 (br m, 2H, **H12**), 1.22 (s, 9H, **H15**) ppm.

^{13}C NMR (100 MHz, DMSO-d_6) δ = 159.4 (**C1**), 155.9 (**C13**), 155.4 (**C2**), 150.8 (**C3**), 139.7 (**C4**), 135.1 (**C5**), 134.6 (**C6**), 133.2 (**C7**), 130.9 (**C8**), 121.2 (**C9**), 120.6 (**C10**), 78.1 (**C14**), 44.6 (**C11**), 36.8 (**C12**), 28.0 (**C15**) ppm.

HRMS (ESI) m/z: $[M + H]^+$ Calcd for $C_{17}H_{18}O_4N_5Br_2$ 513.9726; Found 513.9739.

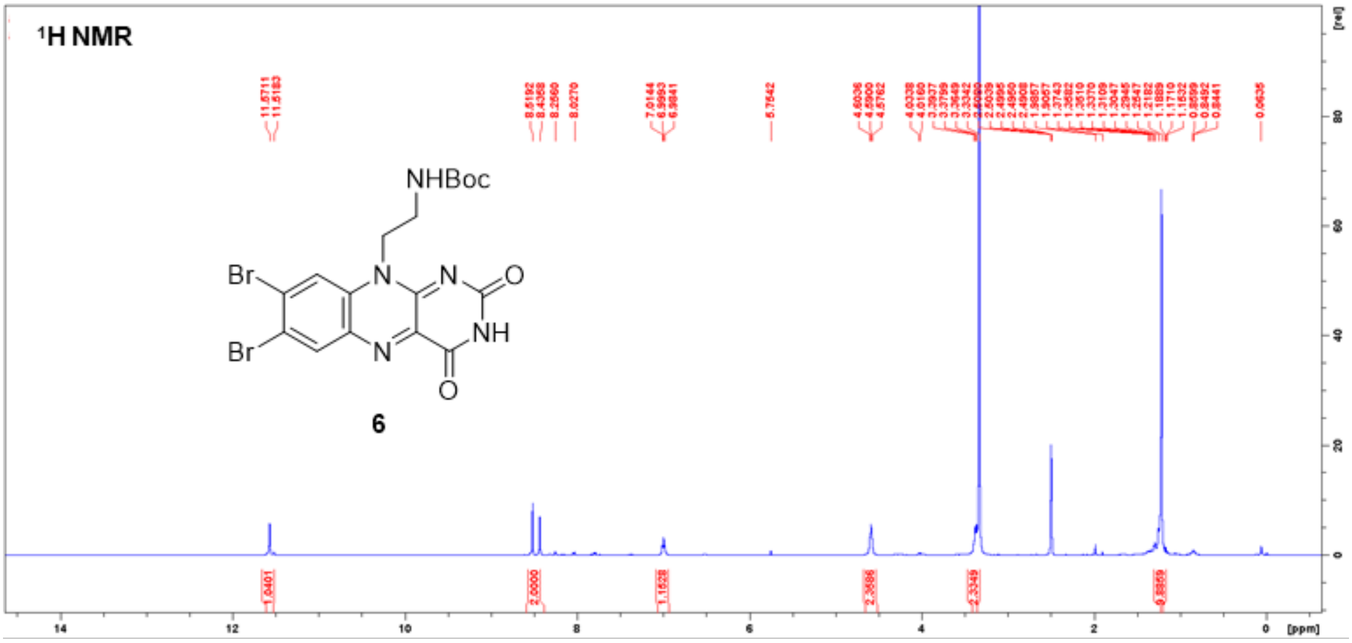

1H NMR
NHBoc
Br
Br
N
N
N
NH
O
O
6

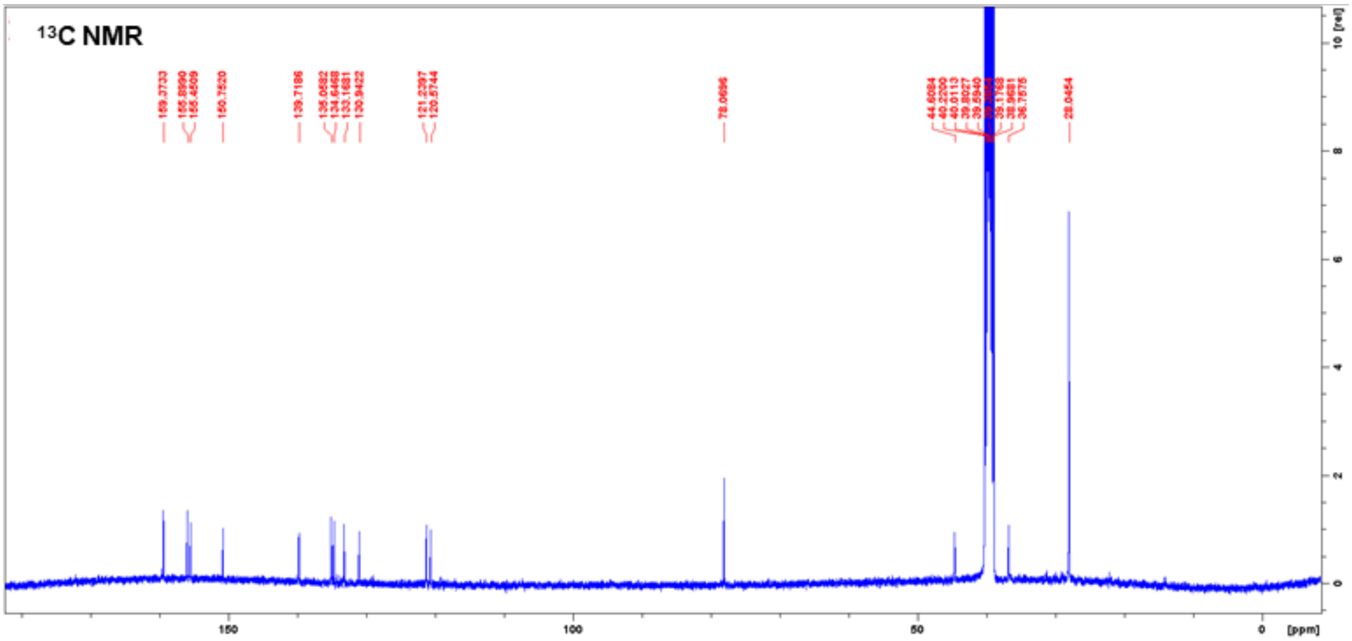

13C NMR

***tert*-Butyl (2-(7,8-dibromo-3-octyl-2,4-dioxo-3,4-dihydrobenzo[*g*]pteridin-10(2*H*)-yl)ethyl)carbamate (7):**

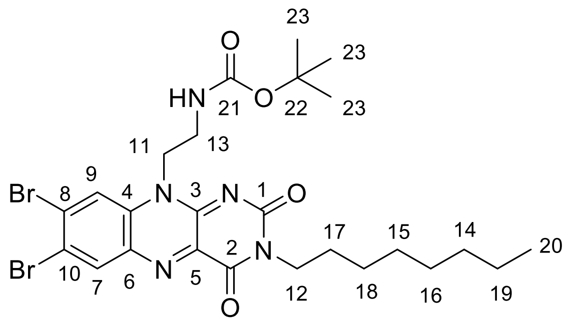

Flavin **6** (504 mg, 0.98 mmol, 1.0 equiv.) and 1-bromooctane (944 mg, 0.86 mL, 4.89 mmol, 5.0 equiv.) were dissolved in anhydrous DMF (20 mL) under Ar atmosphere. Cs_2CO_3 (478 mg, 1.47 mmol, 1.5 equiv.) was then added and the resulting mixture was stirred for 18 h at RT under Ar in the dark. The solvent was then removed under reduced pressure and the resulting residue was suspended in DCM (20 mL) and washed with water (3 x 20 mL) and brine (20 mL). The organic phase was then dried over Na_2SO_4, filtered, and concentrated under reduced pressure. The resulting residue was purified by silica gel column chromatography (10-20% EtOAc/DCM) to yield flavin **7** as an orange solid (444 mg, 72%).

^{1}H NMR (400 MHz, $CDCl_3$) δ = 8.35 (s, 2H, **H7**, **H9**), 5.27 (br t, *J* = 5.7 Hz, 1H, -**NH**-), 4.72 (t, *J* = 6.2 Hz, 2H, **H12**), 4.01 (t, *J* = 7.6 Hz, 2H, **H11**), 3.67 (q, *J* = 6.0 Hz, 2H, **H13**), 1.65 (quint, *J* = 7.4 Hz, 2H, **H17**), 1.40 (s, 9H, **H23**), 1.36-1.24 (m, 10H, **H14/15/16/18/19**), 0.85 (t, *J* = 6.8 Hz, 3H, **H20**) ppm.

^{13}C NMR (100 MHz, $CDCl_3$) δ = 158.7 (**C1**), 156.6 (**C21**), 154.9 (**C2**), 148.6 (**C3**), 137.5 (**C4**), 135.8 (**C5**), 134.9 (**C6**), 133.7 (**C7**), 132.8 (**C8**), 122.8 (**C9**), 120.5 (**C10**), 80.5 (**C22**), 44.7 (**C11**), 42.4 (**C12**), 38.3 (**C13**), 31.8 (**C14**), 29.3 (**C15**), 29.2 (**C16**), 28.4 (**C23**), 27.7 (**C17**), 27.0 (**C18**), 22.6 (**C19**), 14.1 (**C20**) ppm.

HRMS (ESI) m/z: $[M + H]^+$ Calcd for $C_{25}H_{34}O_4N_5Br_2$ 626.0978; Found 626.0980.

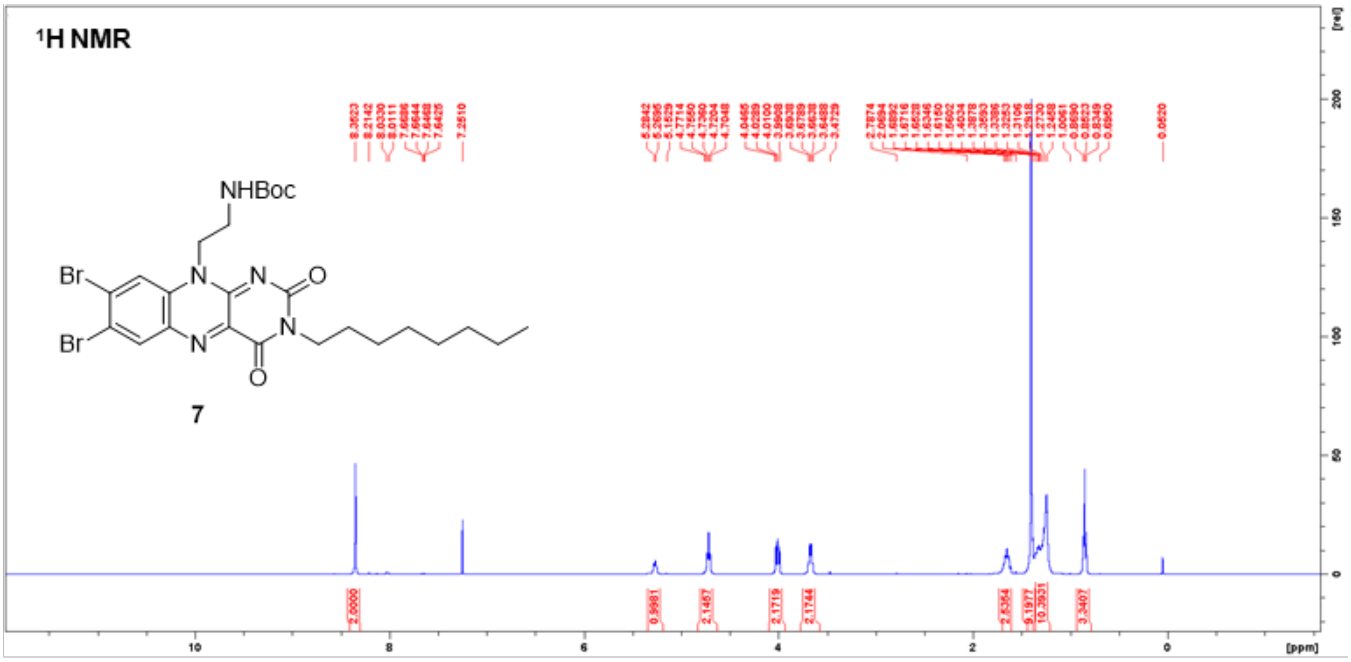
1H NMR
NHBoc
Br
Br
N
N
N
N
O
O
7
[ppm]

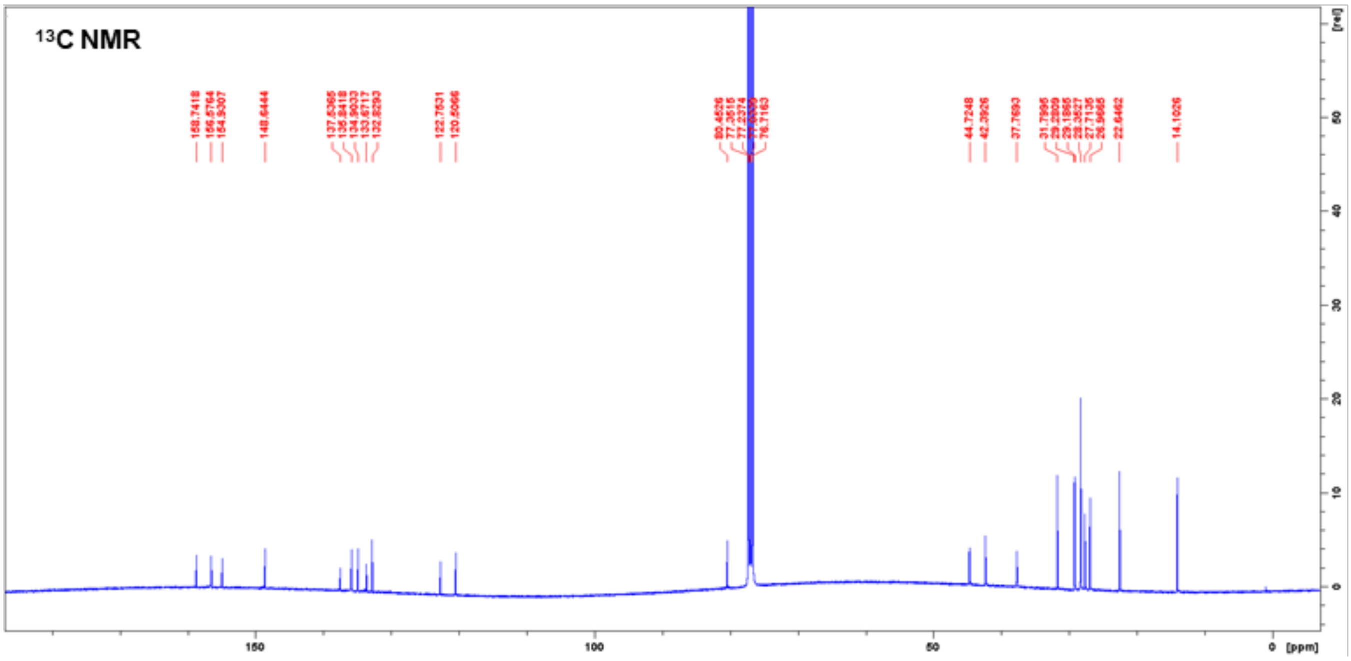
13C NMR
150
100
50
0
[ppm]

2-(7,8-Dibromo-3-octyl-2,4-dioxo-3,4-dihydrobenzo[g]pteridin-10(2H)-yl)ethan-1-aminium trifluoroacetate salt (F3):

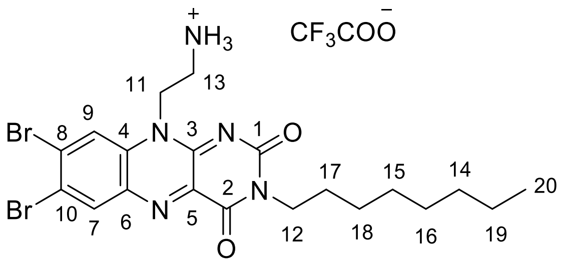

Flavin **7** (212 mg, 0.34 mmol) was dissolved in DCM (4 mL) and TFA (1.5 mL) was added dropwise. The resulting mixture was stirred at RT for 2 h (reaction completion judged by TLC) before the solvent was removed under reduced pressure. Excess TFA was removed using toluene (20 mL) and DCM (3 x 20 mL) co-evaporation to yield the TFA salt of flavin **F3** as a yellow solid (215 mg, 99%).

^{1}H NMR (400 MHz, DMSO-d$_6$) δ = 8.61 (s, 1H, **H7**), 8.35 (s, 2H, **H9**), 7.93 (br m, *J* = 5.7 Hz, 2H, -**NH$_2$**), 4.82 (t, *J* = 6.6 Hz, 2H, **H12**), 3.88 (t, *J* = 7.4 Hz, 2H, **H11**), 3.67 (br m, 2H, **H13**), 1.57 (quint, *J* = 7.3 Hz, 2H, **H17**), 1.30-1.23 (m, 10H, **H14/15/16/18/19**), 0.86 (t, *J* = 6.9 Hz, 3H, **H20**) ppm.

^{13}C NMR (100 MHz, DMSO-d$_6$) δ = 158.9 (**C1**), 154.8 (**C2**), 150.1 (**C3**), 139.3 (**C4**), 135.4 (**C5**), 135.0 (**C6**), 132.3 (**C7**), 131.6 (**C8**), 121.1 (**C9**), 120.8 (**C10**), 41.4 (**C11**), 41.3 (**C12**), 36.4 (**C13**), 31.4 (**C14**), 28.9 (**C15**), 28.8 (**C16**), 27.5 (**C17**), 26.6 (**C18**), 22.3 (**C19**), 14.1 (**C20**) ppm.

HRMS (ESI) m/z: $[M + H]^+$ Calcd for $C_{20}H_{26}O_2N_5Br_2$ 526.0453; Found 526.0455.

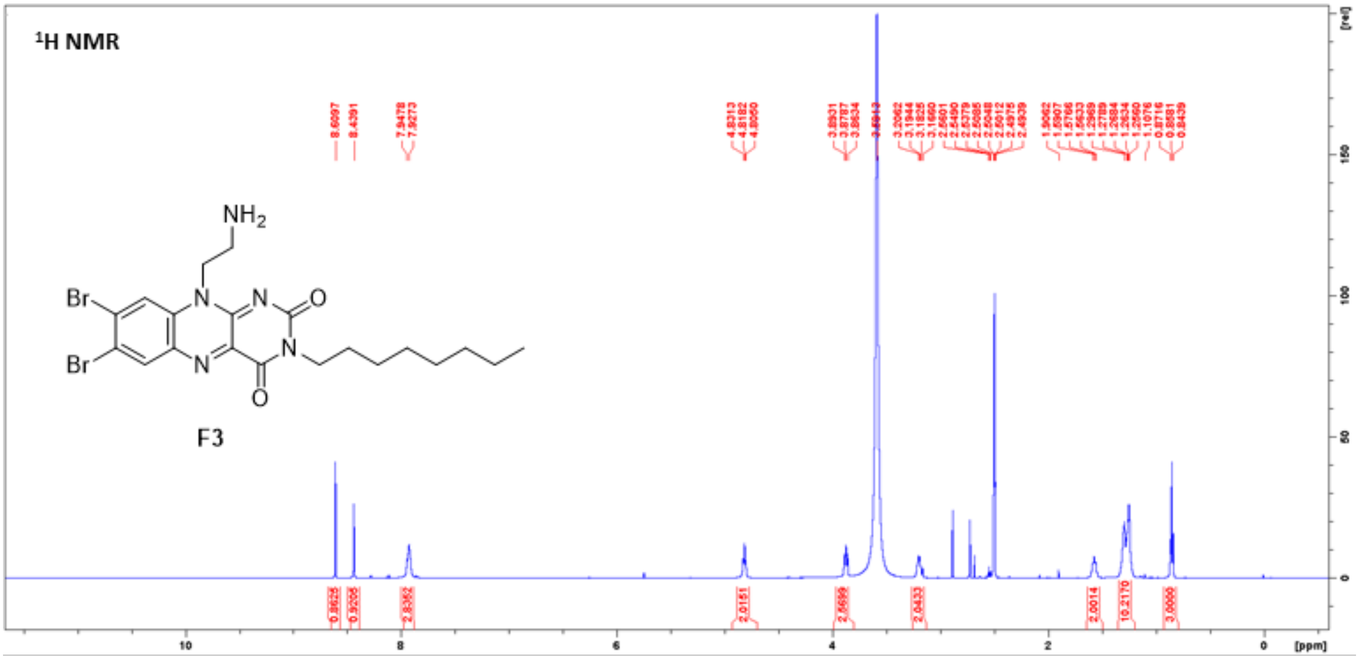
1H NMR
NH2
Br
Br
N
N
N
N
O
O
F3
[ppm]
[rel]

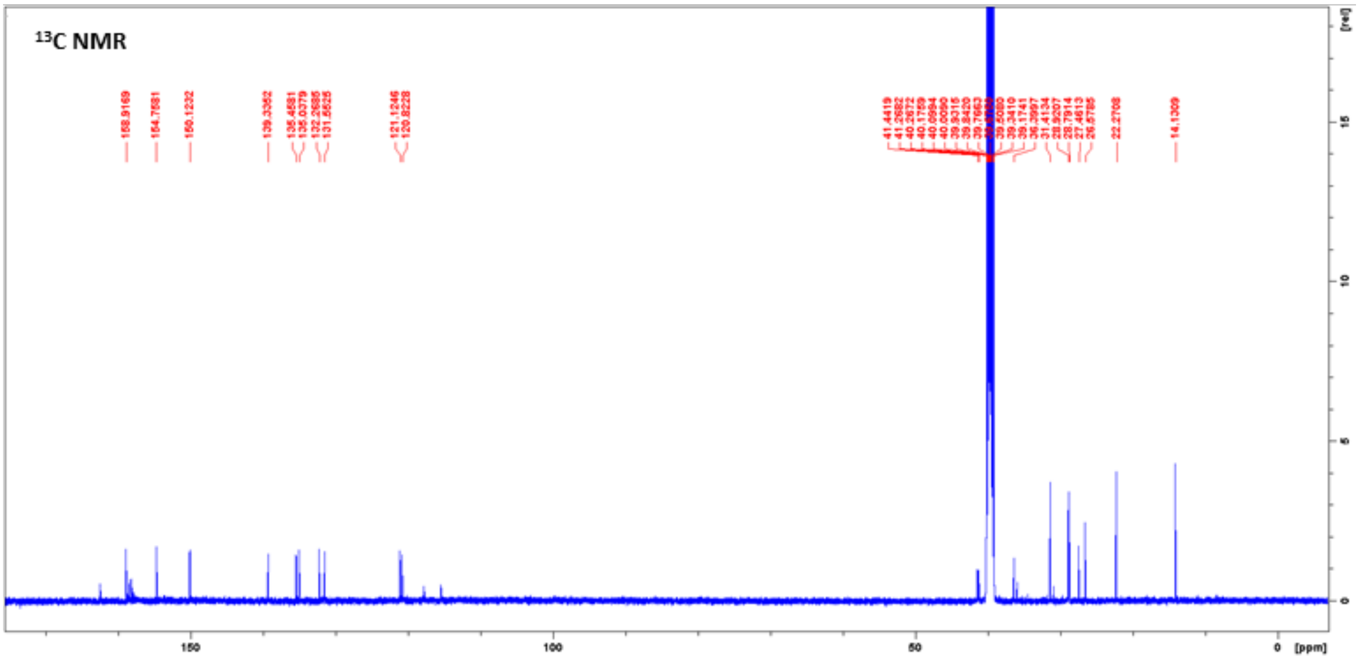
13C NMR
[ppm]
[rel]

1,3-Di-Boc-2-(2-(7,8-dibromo-3-octyl-2,4-dioxo-3,4-dihydrobenzo[g]pteridin-10(2H)-yl)ethyl)guanidine (8):

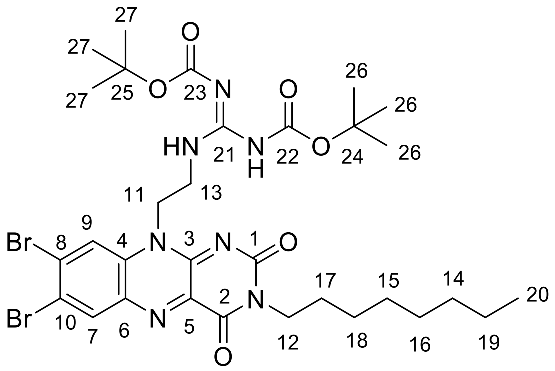

Flavin **7** (270 mg, 0.43 mmol) was dissolved in DCM (4 mL) and TFA (1.5 mL) was added dropwise. The resulting mixture was stirred at RT for 2 h (reaction completion judged by TLC) before the solvent was removed under reduced pressure. Excess TFA was removed using toluene (3 x 20 mL) and DCM (3 x 20 mL) co-evaporation to yield flavin **3** which was then dissolved in anhydrous DMF (5 mL) under Ar atmosphere. DIPEA (0.15 mL, 0.86 mmol, 2.0 equiv.) was then added, followed by *N*,*N'*-di-Boc-1*H*-pyrazole-1-carboxamidine (133 mg, 0.43 mmol, 1.0 equiv). The mixture was then stirred for 18 h at RT under Ar atmosphere in the dark. The solvent was then removed under reduced pressure and the resulting residue was suspended in DCM (20 mL) and washed with water (3 x 20 mL) and brine (20 mL). The organic phase was then dried over Na_2SO_4, filtered, and concentrated under reduced pressure. The resulting residue was purified by silica gel column chromatography (10% EtOAc/DCM) to yield flavin **8** as an orange solid (264 mg, 80% over two steps).

^{1}H NMR (400 MHz, $CDCl_3$) δ = 11.3 (br s, 1H, -**NH**-), 8.57 (br t, *J* = 5.8 Hz 1H, **-NH**-), 8.45 (s, 1H, **H7**), 8.24 (s, 1H, **H9**), 4.88 (t, *J* = 6.0 Hz, 2H, **H12**), 4.04 (t, *J* = 7.6 Hz, 2H, **H11**), 3.88 (q, *J* = 6.1 Hz, 2H, **H13**), 1.68 (quint, *J* = 7.5 Hz, 2H, **H17**), 1.46 (s, 9H, **H26**), 1.43 (s, 9H, **H27**), 1.40-1.21 (m, 10H, **H14/15/16/18/19**), 0.85 (t, *J* = 7.0 Hz, 3H, **H22**) ppm.

^{13}C NMR (100 MHz, $CDCl_3$) δ = 162.9 (**C21**), 158.8 (**C1**), 156.8 (**C23**), 155.0 (**C2**), 152.8 (**C22**), 148.9 (**C3**), 137.9 (**C4**), 136.2 (**C5**), 134.9 (**C6**), 133.0 (**C7**), 132.7 (**C8**), 122.2 (**C9**), 120.4 (**C10**), 83.8 (**C24**), 79.9 (**C25**), 43.6 (**C11**), 42.3 (**C12**), 37.6 (**C13**), 31.8 (**C14**), 29.3 (**C15**), 29.2 (**C16**), 28.3 (**C26**), 28.0 (**C27**), 27.7 (**C17**), 26.9 (**C18**), 22.6 (**C19**), 14.1 (**C20**) ppm.

HRMS (ESI) m/z: $[M + H]^+$ Calcd for $C_{31}H_{44}O_6N_7Br_2$ 768.1720; Found 768.1720.

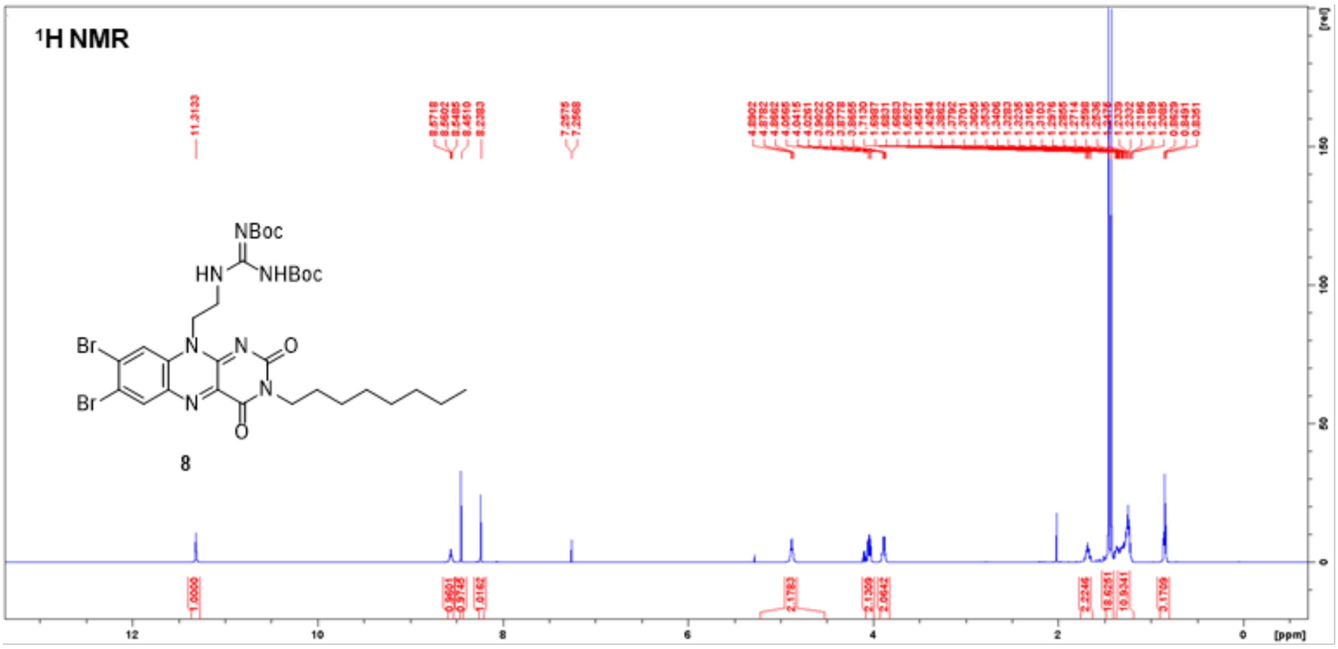
1H NMR
NBoc
HN
NHBoc
Br
Br
N
N
N
N
O
O
8
[ppm]

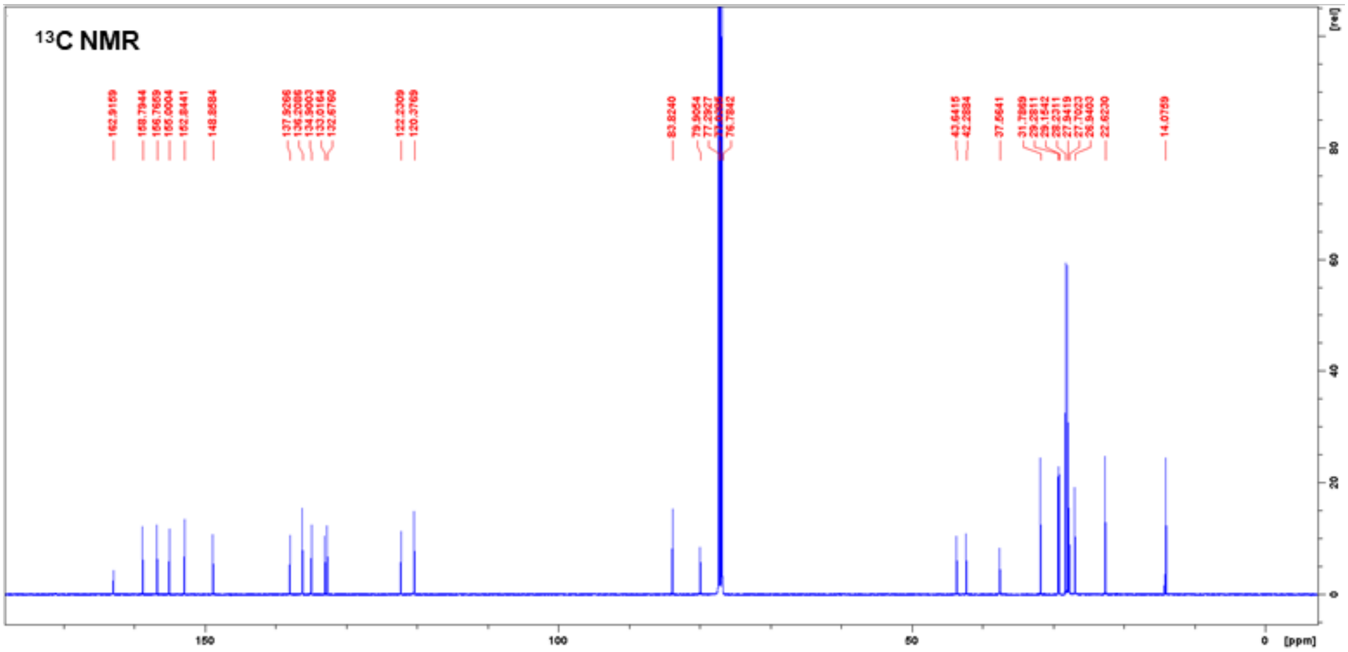
13C NMR
[ppm]

1-(2-(7,8-Dibromo-3-octyl-2,4-dioxo-3,4-dihydrobenzo[g]pteridin-10(2H)-yl)ethyl)guanidinium trifluoroacetate (F4):

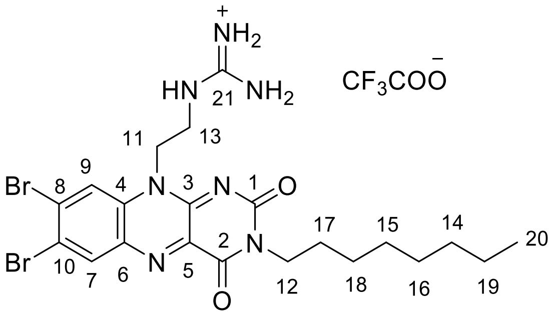

Flavin **8** (264 mg, 0.34 mmol) was dissolved in DCM (4 mL) and TFA (1.5 mL) was added dropwise. The resulting mixture was stirred at RT for 2 h (reaction completion judged by TLC) before the solvent was removed under reduced pressure. Excess TFA was removed using toluene (20 mL) and DCM (3 x 20 mL) co-evaporation to yield the TFA salt of flavin **F4** as a yellow solid (232 mg, 99%).

^{1}H NMR (400 MHz, DMSO-d$_6$) δ = 8.61 (s, 1H, **H7**), 8.42 (s, 1H, **H9**), 7.43 (br t, *J* = 6.3 Hz, 1H, -**NH**-) 4.71 (t, *J* = 6.0 Hz, 2H, **H12**), 3.87 (br s, 3H, =**NH**, -**NH$_2$**), 3.57 (t, *J* = 6.1 Hz, 2H, **H11**), 3.59 (br m, 2H, **H13**), 2.50 (s, 3H, **H20**), 2.41 (s, 3H, **H21**), 1.58 (br m, *J* = 7.42 Hz, 2H, **H17**), 1.33-1.23 (m, 10H, **H14/15/16/18/19**), 0.86 (t, *J* = 6.7 Hz, 3H, **H22**) ppm.

^{13}C NMR (100 MHz, DMSO-d$_6$) δ = 158.9 (**C1**), 157.3 (**C21**), 154.6 (**C2**), 149.5 (**C3**), 139.1 (**C4**), 135.2 (**C5**), 134.9 (**C6**), 132.4 (**C7**), 131.3 (**C8**), 121.0 (**C9**), 120.9 (**C10**), 43.2 (**C11**), 41.1 (**C12**), 37.5 (**C13**), 31.3 (**C14**), 28.8 (**C15**), 28.7 (**C16**), 27.3 (**C17**), 26.5 (**C18**), 22.2 (**C19**), 14.0 (**C20**) ppm.

HRMS (ESI) m/z: [M + H]$^+$ Calcd for $C_{21}H_{28}O_2N_7Br_2$ 568.0671; Found 568.0669.

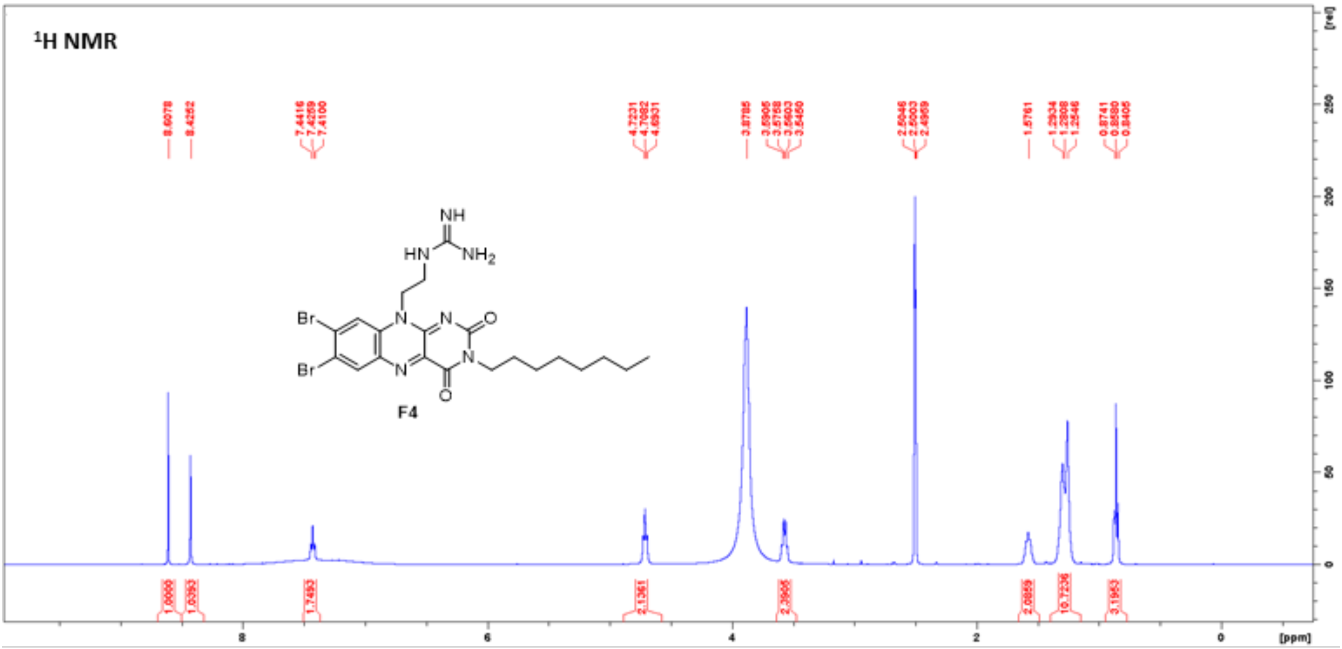
^{1}H NMR
F4
[ppm]

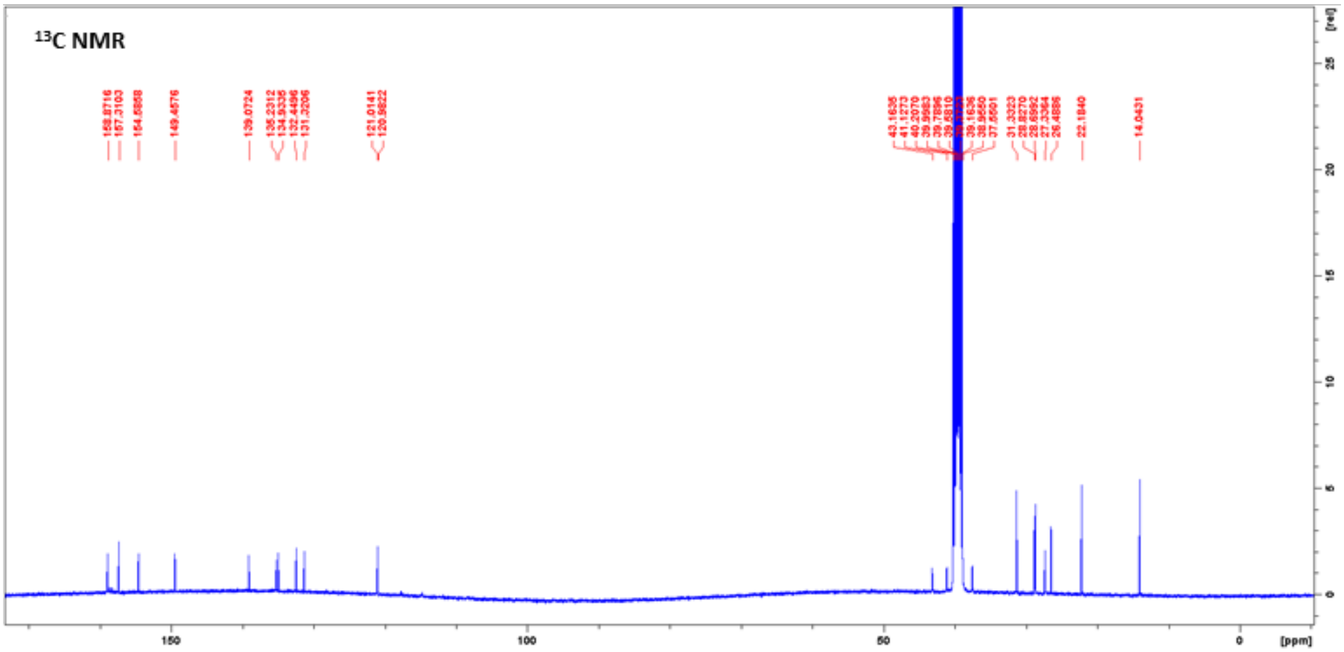
^{13}C NMR
[ppm]

4 Fluorescence Quantum Yield Calculations

The fluorescence quantum yields (Φ_F) for **F1-4** were determined by preparing a series of dilutions in DMSO ranging in absorbance values from 0.02 to 0.10 and excited at their respective absorbance maxima (see **Table S1**).[3] The emission peak was then integrated and plotted against the absorbance value (see **Figure S1**). Riboflavin (from *Eremothecium ashbyii*, ≥98%, Sigma) was chosen as the reference ϕ_F for calculations (Φ_F= 0.226 ± 0.01 in DMSO)[4] according to equation (1):

$$\Phi_{\mathrm{F;Sample}} = \Phi_{\mathrm{F;Ref}} \left(\frac{Slope_{Sample}}{Slope_{Ref}}\right)\left(\frac{\eta^2_{Sample}}{\eta^2_{Ref}}\right) \quad (1)$$

Where Slope = gradient from the plot of integrated emission peak vs. absorption value and η = refractive index of the solvent.

Table S1: Spectroscopic details of flavins **F1-4** and reference compound riboflavin used for ϕ_F calculations in DMSO.

Flavin	ε ($M^{-1}cm^{-1}$)	λ_{ex} (nm)	λ_{em} (nm)
**Riboflavin**	11718.41	447	513
**F1**	10368.79	444	507
**F2**	9838.05	446	512
**F3**	9541.26	443	508
**F4**	10208.50	444	513

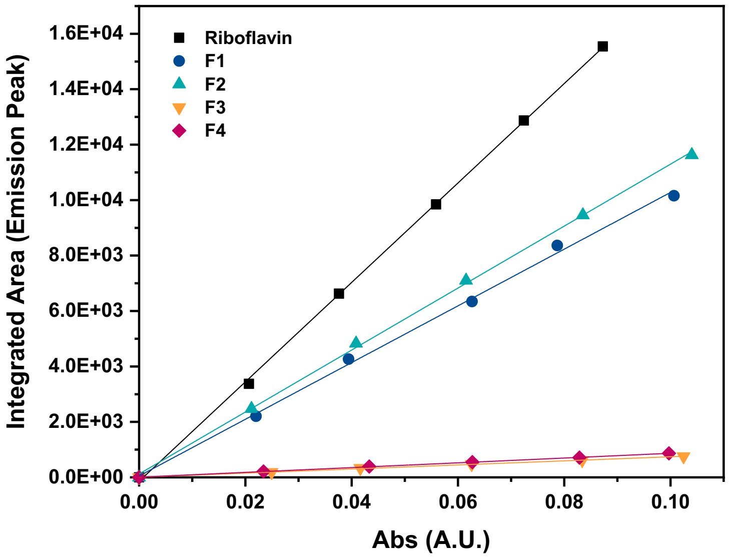

Figure S2: Plot of integrated emission peak against absorbance value of flavins **F1-4** and reference compound riboflavin in DMSO.

5 Singlet Oxygen Quantum Yield Calculations

The singlet oxygen quantum yield (Φ_Δ) of flavins **F1-4** were calculated using the singlet oxygen probe, 1,3-diphenylisobenzofuran (DPBF, 97%, Acros Organics, UK) according to the guidelines published by Bresolí-Obach *et al.*[5] The light source used was a 450 nm LED (18 W, HepatoChem, US) with the light output attenuated using a 2" x 2" Absorptive Neutral Density Filter (OD 3.0, Thor Labs, UK) as to avoid considerable self-bleaching of DPBF (blank control). $Ru(bpy)_3^{2+}$ (dichloro hexahydrate, 99.95%, Sigma-Aldrich, UK) was chosen as the reference ϕ_Δ for calculations ($\Phi_\Delta = 0.57 \pm 0.06$ in MeCN)[6] according to equation (2):

$$\Phi_{\Delta;\mathrm{Sample}} = \Phi_{\Delta;\mathrm{Ref}} \frac{(Slope_{Sample} - Slope_{Control}) \cdot (1 - 10^{-A_{Ref}})}{(Slope_{Ref} - Slope_{Control}) \cdot (1 - 10^{-A_{Sample}})} \left(\frac{\eta^2_{Sample}}{\eta^2_{Ref}} \right) \quad (2)$$

Where Slope = rate of DPBF photobleaching, Sample = flavin **F1-4**, Control = DPBF alone, Ref = $Ru(bpy)_3^{2+}$, A = absorbance value at irradiation wavelength (450 nm) and η = refractive index of the solvent.

General procedure: DPBF (50 µM) was irradiated with stirring at 450 nm in the presence of flavin (20 µM) or $Ru(bpy)_3^{2+}$ (15 µM) in MeCN (3 mL) at RT with the reaction vessel open to air. Aliquots of the reaction (100 µL) were taken every 30 s for 3 min and measured by UV-Vis spectroscopy. The decrease of the λ_{max} of DBPF (410 nm) was then evaluated and plot according to A/A_0 vs. time as shown below (**Figure S2**). Experiments were carried out three separate times using fresh stock solutions of the reagents. The experimental error was estimated by combining the $\Phi_{\Delta;Ref}$ error and the standard deviation of the average Φ_Δ for the flavin samples.

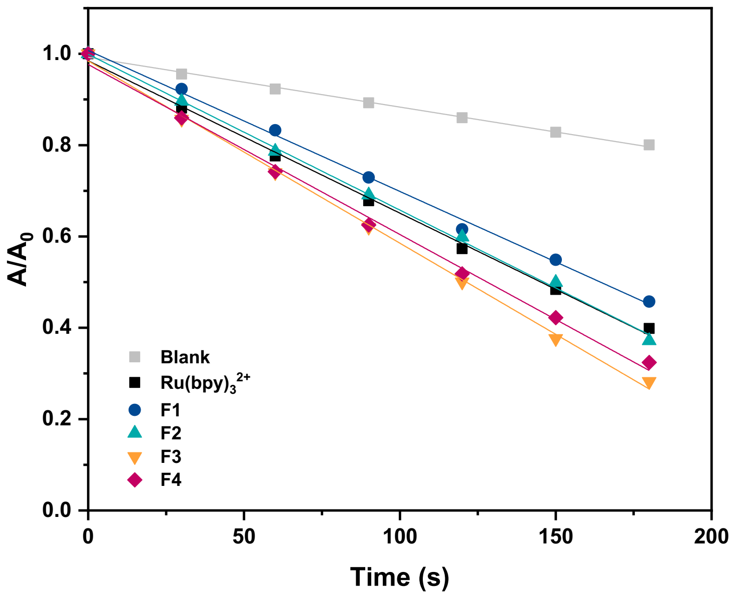

Figure S3: Plot of DPBF bleaching in the presence of flavins **F1-4** or reference compound $Ru(bpy)_3^{2+}$ under 450 nm irradiation in MeCN. Blank = DPBF alone with no photosensitiser.

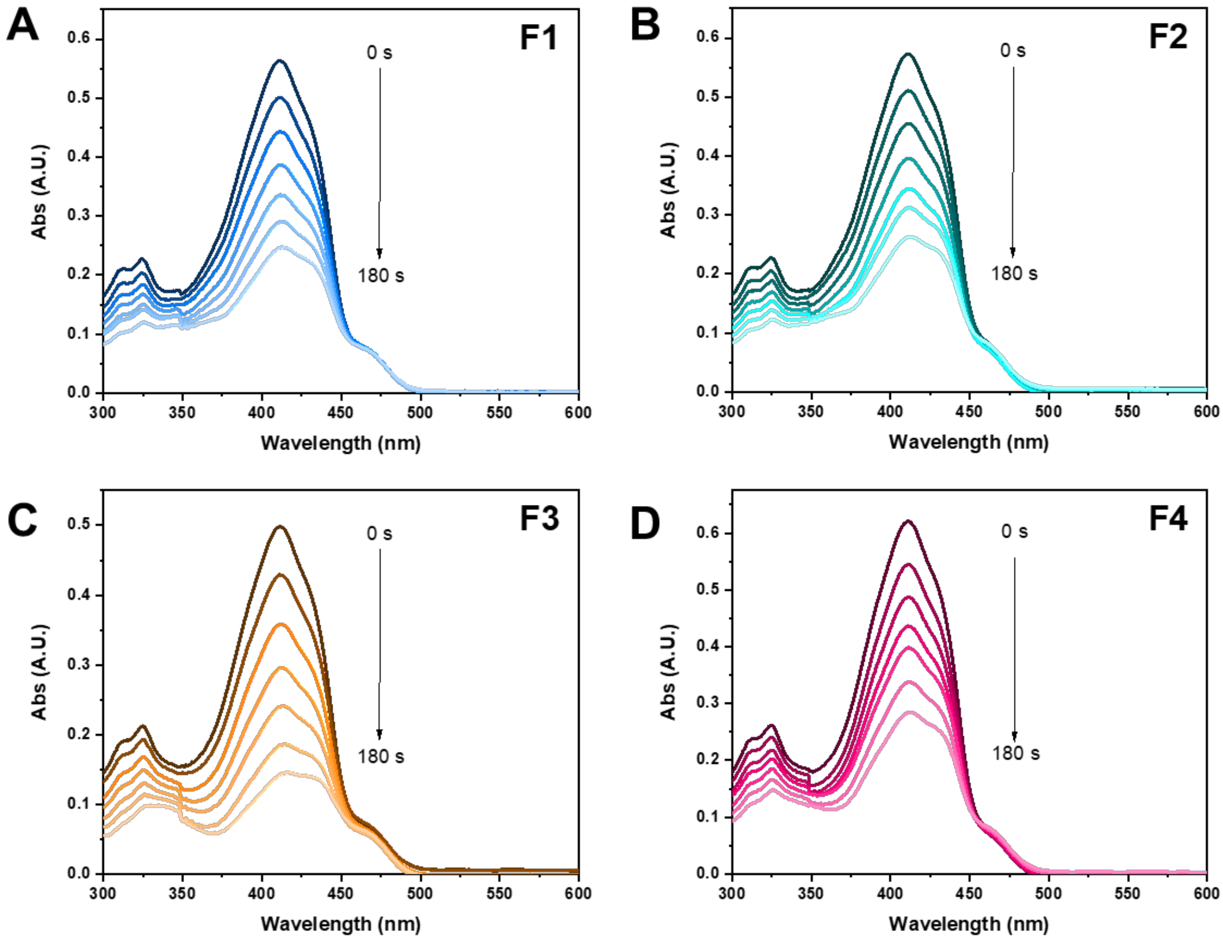

Figure S4: UV-Vis absorption spectra of DPBF bleaching in the presence of flavins **F1-4** under 450 nm irradiation in MeCN.

6 Flavin Photostability

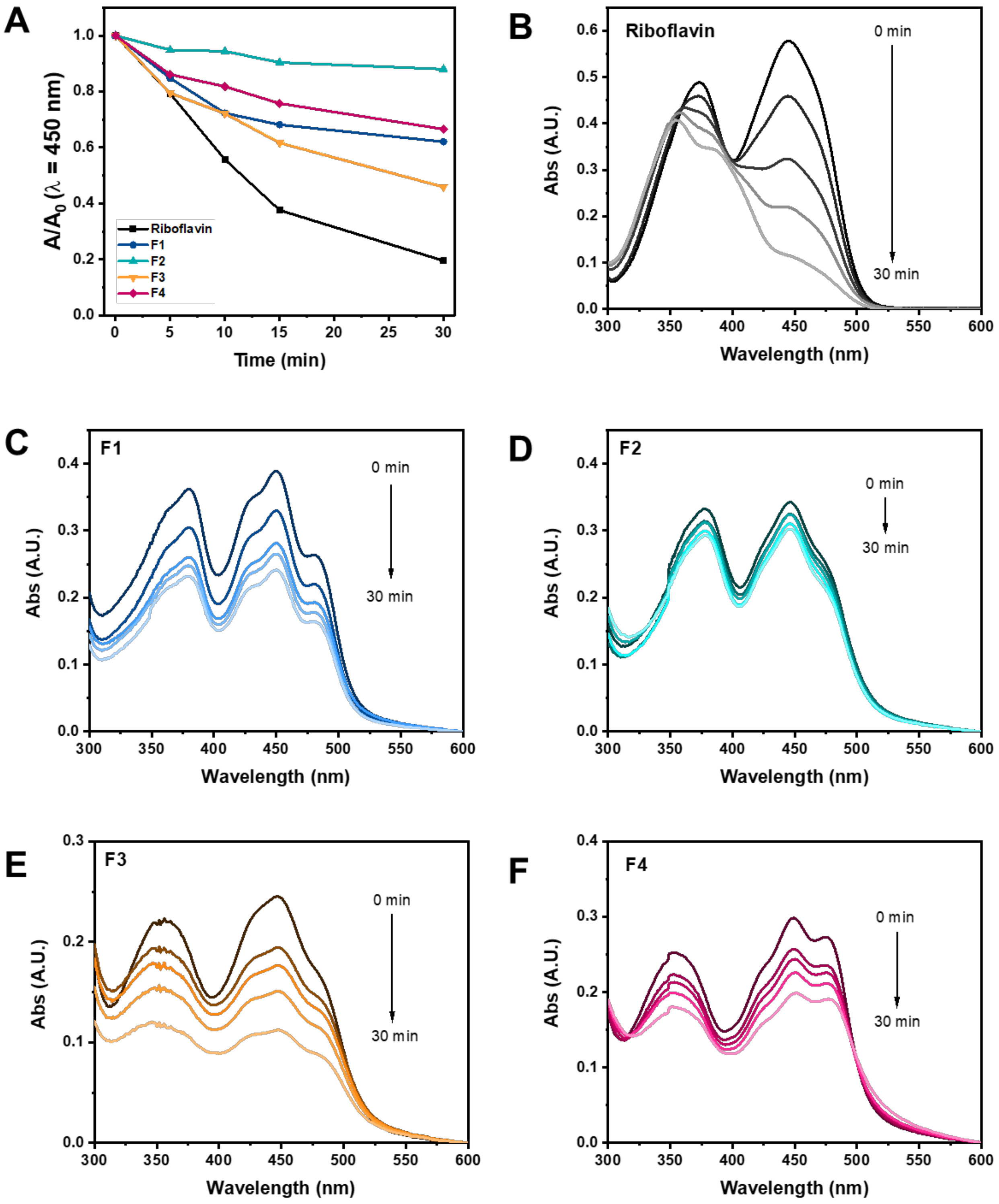

Figure S4: (A) Flavin photostability (100 µM) in PBS (0.1% DMSO) irradiated with 1x10^5 lx (35 mW/cm^2) where A = Abs at 450 nm and A_0 = Abs at 450 nm at 0 min. (B-F) Associated UV-Vis spectra of riboflavin and **F1-4** photostability. Experiments were performed using a quartz cuvette and measured using an Agilent Cary 300 Spectrophotometer.

7 Bacterial Culture and Phototoxicity Assay

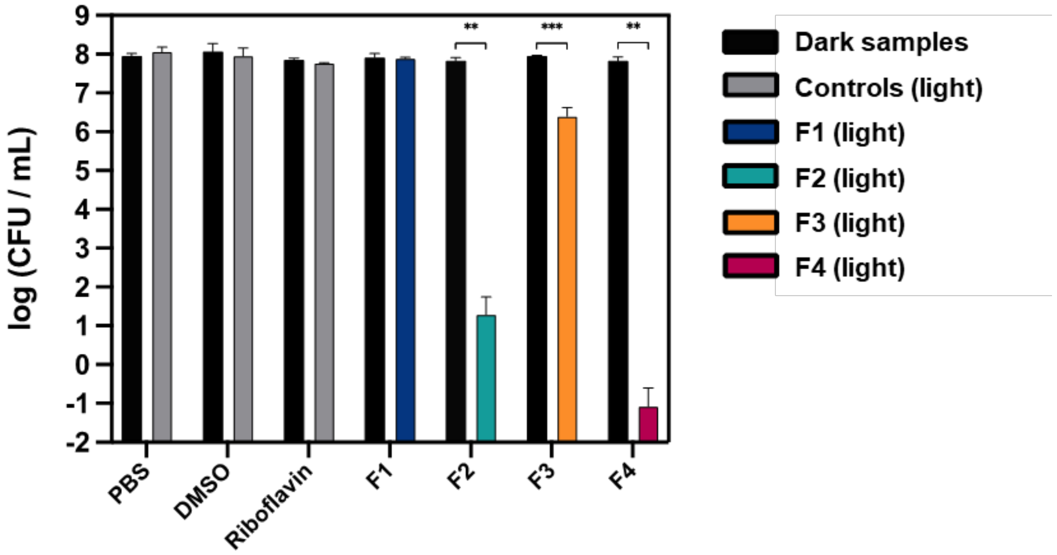

Figure S5: Photodynamic inactivation of *E. coli* incubated with 100 µM of flavin compound irradiated with white LED light (1x10^5 lx, 35 mW/cm^2) or incubated in the dark for 15 min in PBS (0.5% DMSO). Data are expressed as the mean ± SD of three biological replicates. Significance levels are defined as the following: ns for $p>0.05$, * for $p\leq0.05$, ** for $p\leq0.01$, *** for $p<0.001$, and **** for $p<0.0001$.

7.1 *E. coli* Log_{10} *Reductions*

Table S2: Table of *E. coli* log_{10} reductions in the presence of flavin under irradiation.

Flavin	Irradiation time (min)	Concentration (µM)	Log_{10} reduction[a]
**Riboflavin**	15	1	0.2
	15	10	0.2
	5	100	0.0
	10	100	0.0
	15	100	0.0
**F1**	15	1	0.2
	15	10	0.2
	5	100	0.1
	10	100	0.1
	15	100	0.1
**F2**	15	1	0.2
	15	10	0.8
	5	100	3.4
	10	100	5.9
	15	100	7.0
**F3**	15	1	0.2
	15	10	0.4
	5	100	1.3
	10	100	1.7
	15	100	1.8
**F4**	15	1	0.2
	15	10	2.8
	1	100	4.1[b]
	2	100	5.8[b]
	3	100	6.4[b]
	4	100	7.6[b]
	5	100	8.4
	10	100	8.7
	15	100	9.1

[a] calculated by log_{10}(control) - log_{10}(sample) at respective irradiation time

[b] calculated by $log_{10}(t_0) - log_{10}(t_{sample})$ where $t_0 = 7.9$

7.2 SIM Imaging

Structured illumination microscopy (SIM) of *E. coli* BL21(DE3) was performed as described previously by Mela *et al.*[7] In detail, a culture of *E. coli* BL21(DE3) was grown overnight at an OD_{600} of ~1. Prior to SIM imaging the cultures were centrifuged at 3000 rpm for 5 minutes and resuspended in PBS (1x. pH 7.4) to an OD_{600} of around 0.5. The *E. coli* suspension was incubated for 1 hour with 100 µM of each one of the flavins (riboflavin, **F1** or **F2** in PBS). The bacteria were then centrifuged at the same settings and the bacterial pellet resuspended in PBS (1x. pH 7.4) to remove excess flavin. 2 µl of the sample were deposited on a glass coverslip and an agarose pad was positioned over the sample to prevent the bacteria from moving during imaging. Another coverslip was positioned on top to minimise drying of the agarose pads.

Images of the sample were collected using 3-color SIM for optical sectioning. A ×60/1.2 NA water immersion lens (UPLSAPO 60XW, Olympus) focused the structured illumination pattern onto the sample, and the same lens was also used to capture the fluorescence emission light before imaging onto an sCMOS camera (C11440, Hamamatsu). The wavelength used for excitation was 488 nm (iBEAM-SMART-488, Toptica). Images were acquired using custom SIM software described previously.[8]

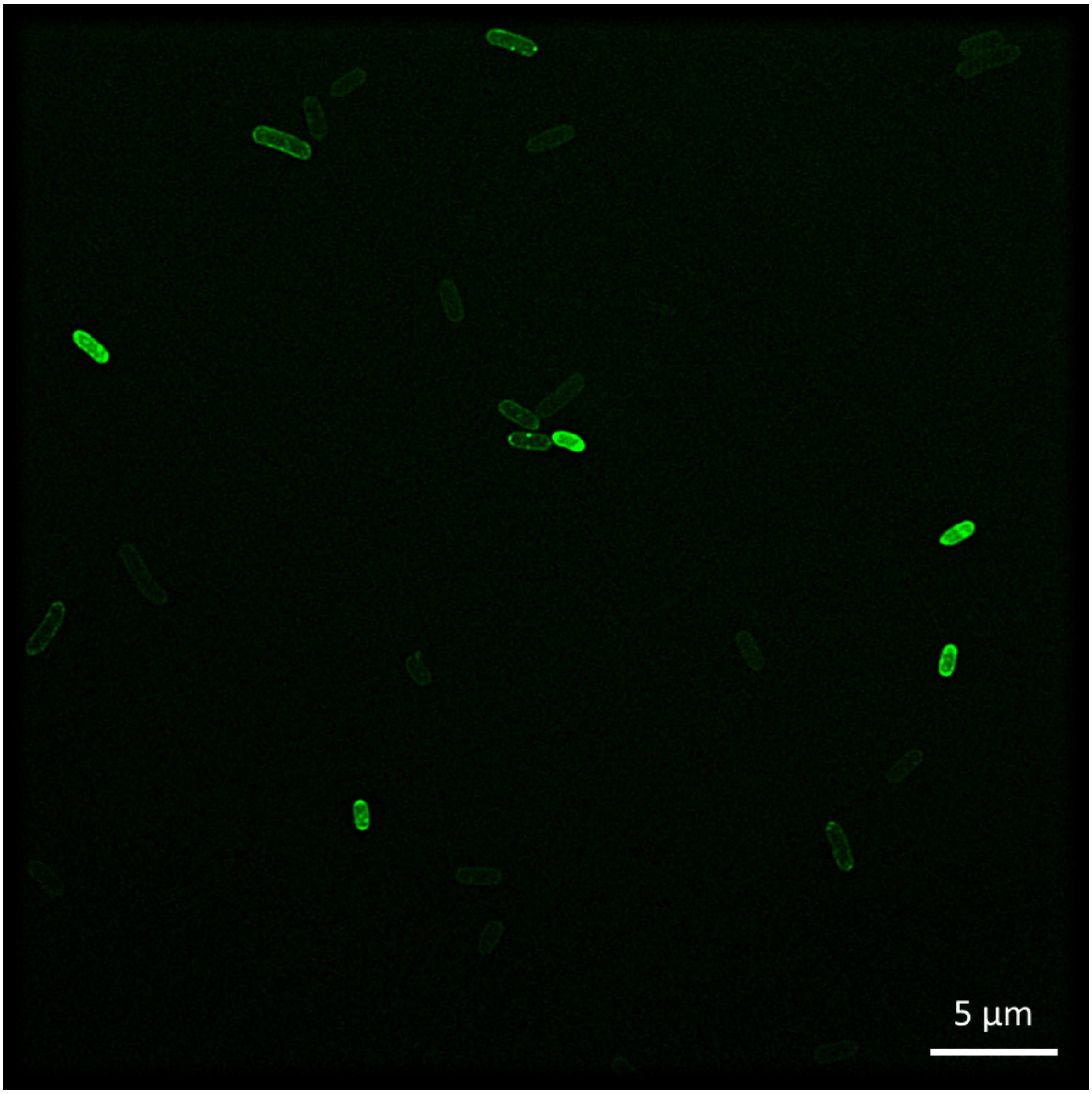

Figure S6: Large field of view (42.8 x 42.8 µm) of *E. coli* after incubation with **F2** (100 µM).

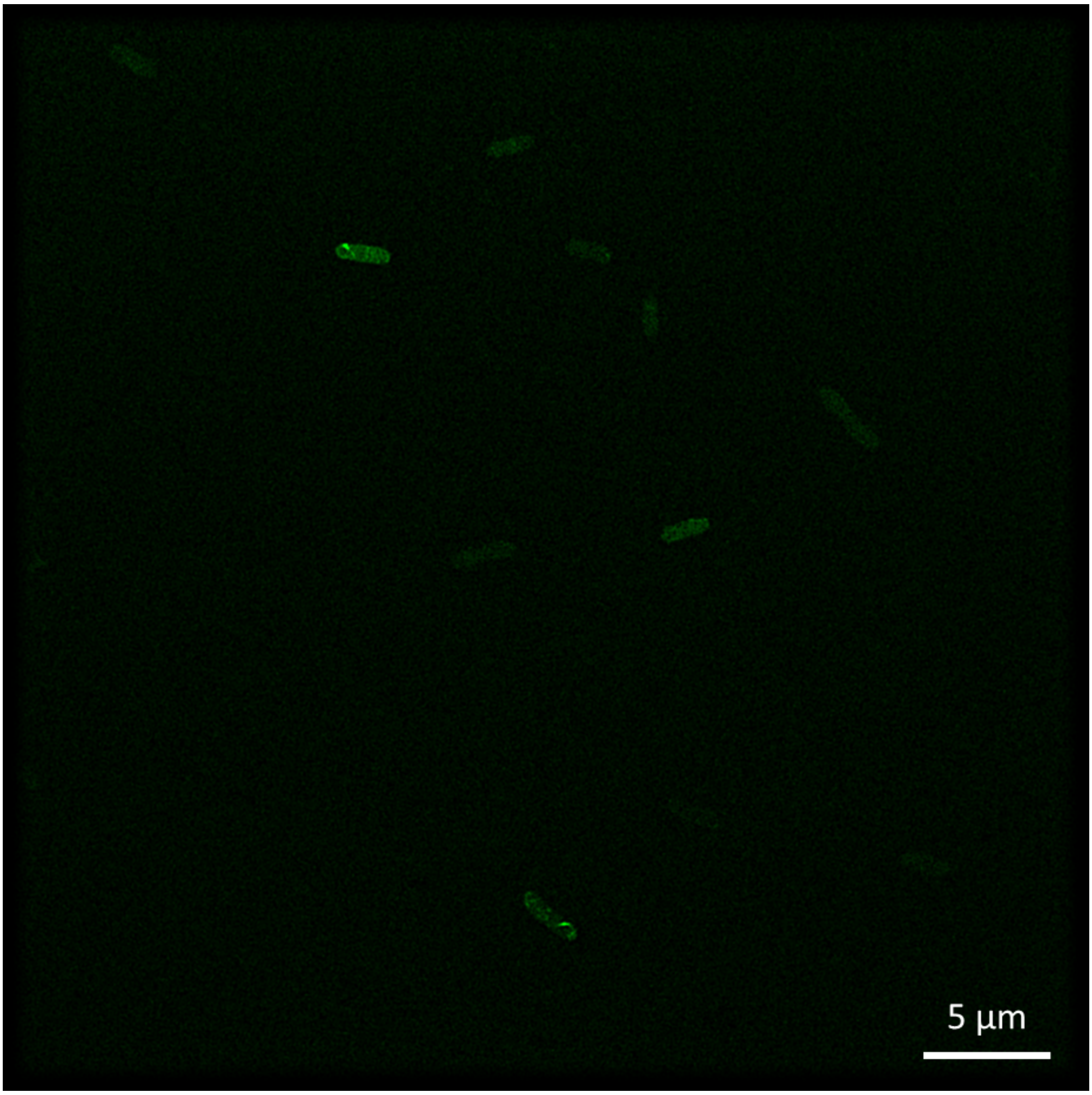

Figure S7: Large field of view (42.8 x 42.8 µm) of *E. coli* after incubation with **F1** (100 µM).

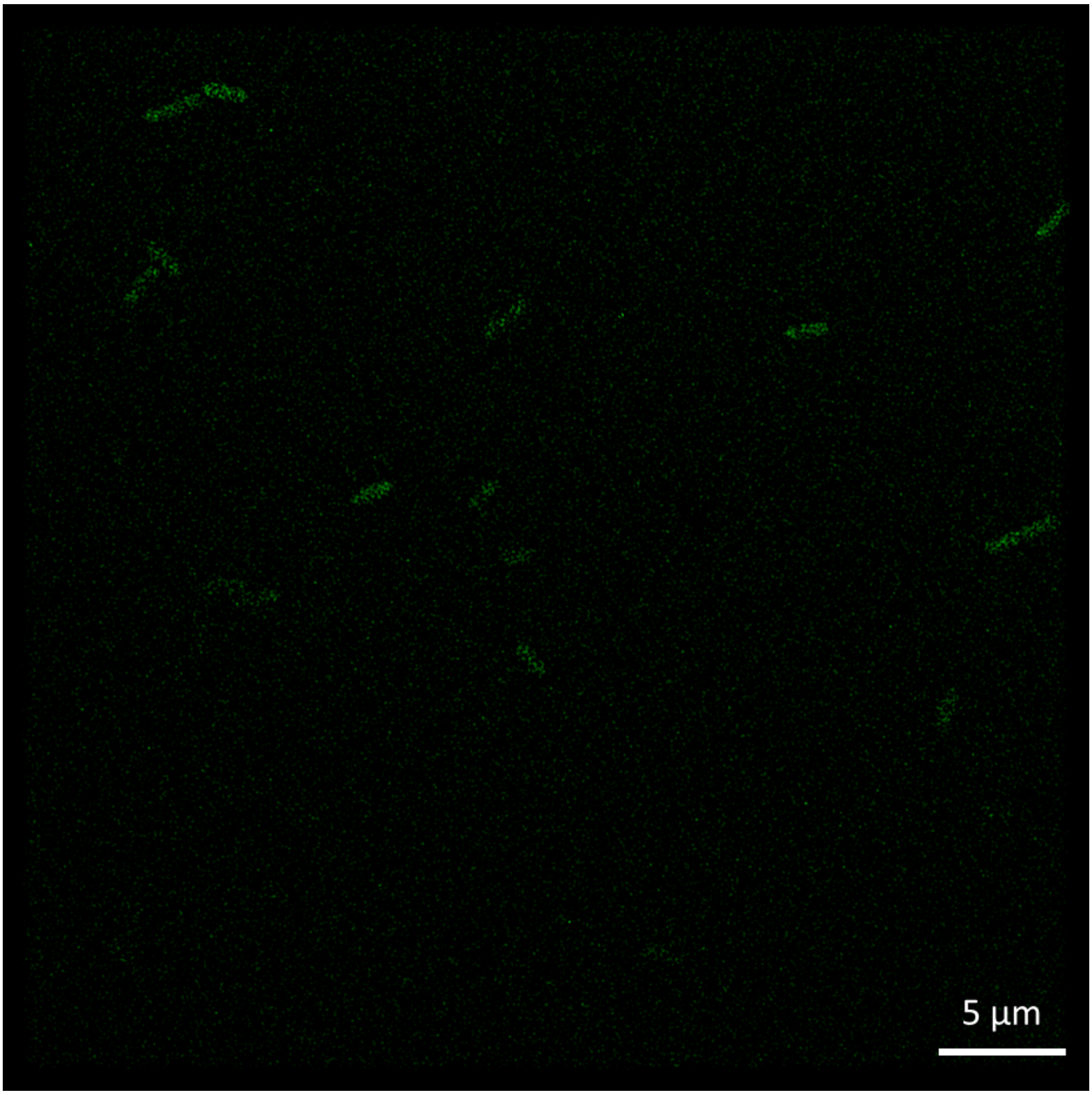

Figure S8: Large field of view (42.8 x 42.8 µm) of *E. coli* after incubation with riboflavin (100 µM).

Gel Electrophoresis

Agarose gel electrophoresis of pDNA (pUC18) following incubation with 10 µM flavin irradiated with white LED light ($1x10^5$ lx, 35 mW/cm^2) for 15 min in PBS containing 0.1% DMSO. Rbf = Riboflavin.

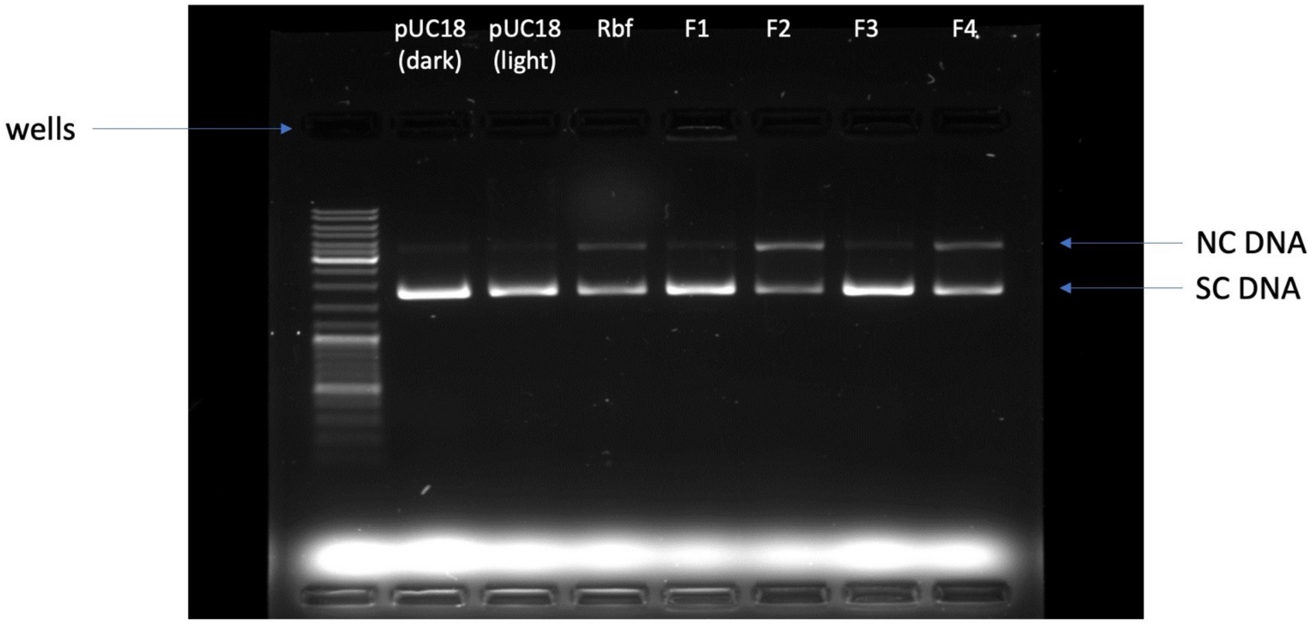

Figure F9: Uncropped agarose gel demonstrating the DNA cleavage as shown in Fig.3d.

Virology and $TCID_{50}$ Assay

7.3 MHV-A59 Log_{10} Reductions

Table S3: Table of MHV-A59 log_{10} reductions in the presence of flavin under irradiation.

Flavin	Irradiation time (min)	Concentration (μM)	Log_{10} reduction[a]
**Riboflavin**	10	1	1.2
	10	5	3.2
	5	10	2.7
	10	10	3.4
	15	10	3.7
**F1**	10	1	0.0
	10	5	1.7
	5	10	1.2
	10	10	2.1
	15	10	2.2
**F2**	10	1	2.1
	10	5	3.9
	5	10	3.0
	10	10	3.8
	15	10	5.0
**F3**	10	1	2.1
	10	5	4.1
	5	10	3.9
	10	10	4.8
	15	10	5.4
**F4**	10	1	3.4
	10	5	4.6
	5	10	3.8
	10	10	5.9
	15	10	6.7

[a] calculated by log_{10}(control) - log_{10}(sample) at respective irradiation time

Gel electrophoresis of ssRNA cleavage

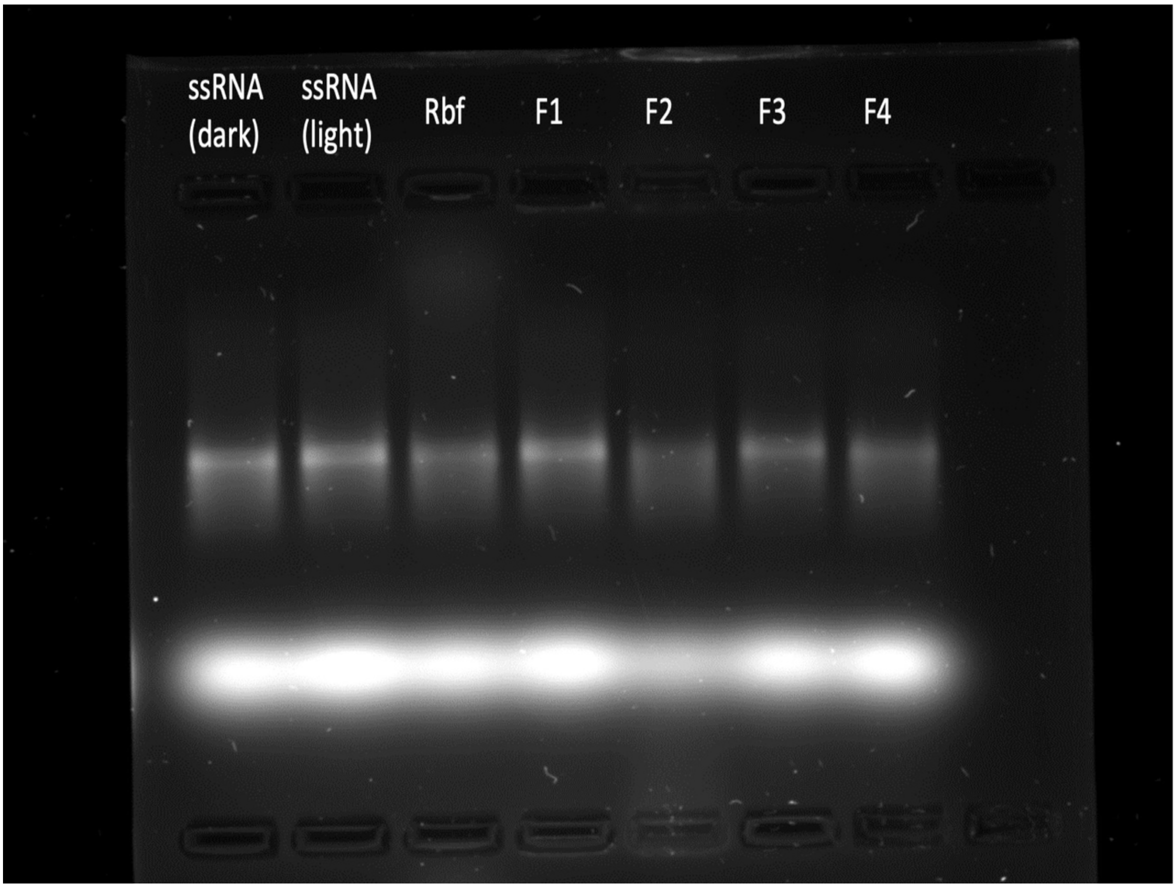

Figure S10: Uncropped gel electrophoresis image of ssRNA cleavage shown in Fig. 4d.

8 Viability and Phototoxicity Assay

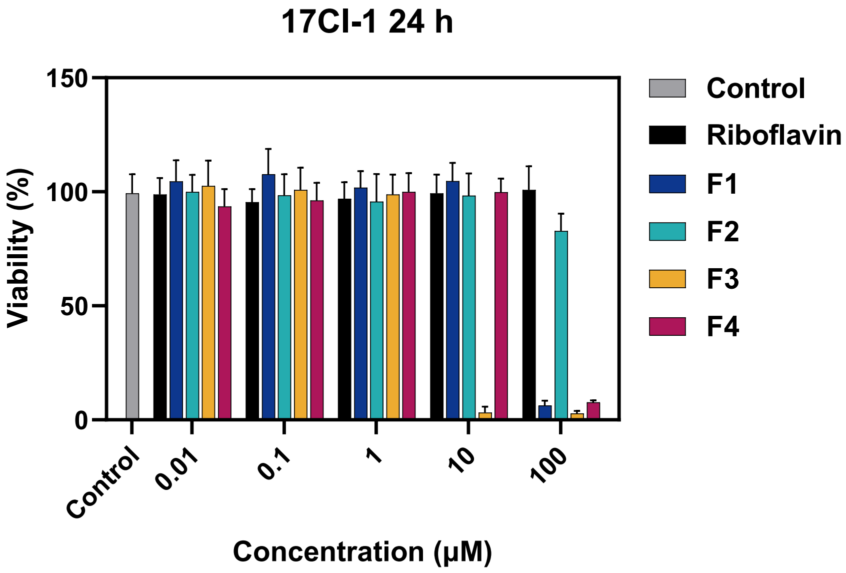

Figure S11. *In vitro* cytotoxicity effect of **F1**–**F4** and riboflavin on 17Cl-1 cells after 24 h incubation determined by MTS assay. Data are expressed as the mean ± SD of three biological replicates.

Table S4: IC_{50} and cLogP values for flavins **F1-4** and riboflavin.

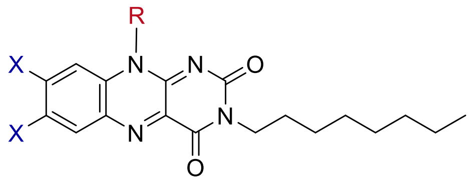

Flavin	X	R	IC_{50} (µM)[a] WI-38	IC_{50} (µM)[a] 17Cl-1	cLogP[b]
**Riboflavin**	Me	Ribityl chain	>100	>100	-2.07
**F1**	Me	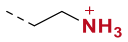	96.1	70.2	-0.55
**F2**	Me	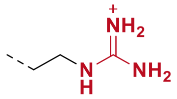	>100	>100	2.59
**F3**	Br	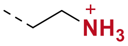	30.9	3.66	0.22
**F4**	Br	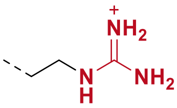	>100	50.9	3.36

[a] determined after 24 h incubation by MTS assay
[b] determined using DataWarrior V5.5.0

9 References

1. Nehme, S. I., Crocker, L. & Fruk, L. Flavin-conjugated iron oxide nanoparticles as enzyme-inspired photocatalysts for Azo Dye degradation. *Catalysts* **10**, 324 (2020).
2. Legrand, Y. M., Gray, M., Cooke, G. & Rotello, V. M. Model Systems for Flavoenzyme Activity: Relationships between Cofactor Structure, Binding and Redox Properties. *J. Am. Chem. Soc.* **125**, 15789–15795 (2003).
3. Pokluda, A. *et al.* Robust Photocatalytic Method Using Ethylene-Bridged Flavinium Salts for the Aerobic Oxidation of Unactivated Benzylic Substrates. *Adv. Synth. Catal.* **363**, 4371–4379 (2021).
4. Drössler, P., Holzer, W., Penzkofer, A. & Hegemann, P. Fluoresence quenching of riboflavin in aqueous solution by methionin and cystein. *Chem. Phys.* **286**, 409–420 (2003).
5. Bresolí-Obach, R., Torra, J., Zanocco, R. P., Zanocco, A. L. & Nonell, S. Singlet oxygen quantum yield determination using chemical acceptors. in *Methods in Molecular Biology* vol. 2202 165–188 (Humana Press Inc., 2021).
6. Abdel-Shafi, A. A., Beer, P. D., Mortimer, R. J. & Wilkinson, F. Photosensitized generation of singlet oxygen from ruthenium(II)-substituted benzoaza-crown-bipyridine complexes. *Phys. Chem. Chem. Phys.* **2**, 3137–3144 (2000).
7. Mela, I. *et al.* DNA Nanostructures for Targeted Antimicrobial Delivery. *Angew. Chemie* **132**, 12798–12802 (2020).
8. Young, L. J., Ströhl, F. & Kaminski, C. F. A guide to structured illumination TIRF microscopy at high speed with multiple colors. *J. Vis. Exp.* **2016**, e53988 (2016).
